# The Gut–Kidney Axis in Chronic Kidney Disease: Microbial Dysbiosis, Host–Microbial Metabolism, and Emerging Therapeutic Strategies

**DOI:** 10.3390/biomedicines14102143

**Published:** 2026-09-22

**Authors:** Louise Vijverman, Too Maddalena, Charlotte Delrue, Marijn M. Speeckaert

**Affiliations:** 1 Department of Nephrology, Ghent University Hospital, 9000 Ghent, Belgium; louise.vijverman@ugent.be (L.V.); too.maddalena@ugent.be (T.M.); charlotte.delrue@ugent.be (C.D.); 2 Research Foundation-Flanders (FWO), 1000 Brussels, Belgium

**Keywords:** chronic kidney disease, gut–kidney axis, gut microbiota, microbial metabolites, microbiome-targeted therapy

## Abstract

Chronic kidney disease (CKD) appears linked to changes in the gut microbiota, intestinal barrier defects, and microbiota-driven changes in metabolite profiles. Experimental evidence links gut microbiota disturbances to kidney injury through pathways such as microbial metabolism, inflammation, immune signals, and epithelial barrier dysfunction. Still, applying this knowledge in clinical practice for patients with CKD remains difficult. For instance, one study on indoxyl sulfate and p-cresyl sulfate levels in a patient showed that, beyond being synthesized by the intestinal microbiota, the host can also metabolize or alter these metabolites. In addition, they undergo impaired renal clearance and can be secreted by the tubules. Moreover, most human microbiome-related studies are observational, making it difficult to determine the causal role of bacterial dysbiosis. Disease, dietary factors, medication, and other comorbid conditions are major confounders. Microbiota-directed means of altering the composition of the microbiota, such as changing diet, using prebiotics, probiotics, synbiotics, oral adsorbents, and even fecal microbiota transplantation, have, to varying degrees, affected the composition of the gut microbiota, levels of uremic toxins, short-chain fatty acids, and markers of inflammation. They have also been connected to leaky gut syndrome. However, evidence that these actions delay eGFR decline or prevent kidney failure, cardiovascular events, hospitalization, or mortality remains limited. Postbiotics and microbiota-derived metabolites in pure form are distinct experimental therapies and need to be assessed independently. This article reviews the gut kidney axis at different levels of evidence and distinguishes preclinical mechanisms, human associations, surrogate biomarker responses, and clinical outcomes. It points out why impressive mechanistic and biomarker results have not translated into better kidney or cardiovascular outcomes. Future research should use standardized methodologies, provide more detail on microbial functions and host metabolite metabolism, and test microbiome-targeted therapies in sufficiently powered randomized controlled trials with meaningful endpoints.

## 1. Introduction

Chronic kidney disease (CKD) remains one of the world’s toughest major public health issues. CKD alone is responsible for cardiovascular disease, premature deaths, and rising medical costs on an ever-growing scale, while over 10 percent of adults worldwide are already suffering from it [1,2]. Despite nephroprotective strategies such as renin–angiotensin–aldosterone system (RAAS) blockers, sodium–glucose cotransporter 2 (SGLT2) inhibitors, and mineralocorticoid receptor antagonists (MRAs), a patient’s kidney function may progressively decline and eventually lead to terminal kidney failure [3,4]. Thus, despite substantial advances in guideline-directed therapy, considerable residual kidney and cardiovascular risk remains, supporting continued investigation of additional pathogenic pathways and complementary therapeutic strategies.

Despite substantial progress in CKD treatment, today’s therapeutic options have major shortcomings. RAAS inhibitors can reduce albuminuria and halt or slow CKD progression. However, they carry risks of hyperkalemia, low blood pressure, and an acute drop in estimated glomerular filtration rate (eGFR), which may limit initiation or dose escalation. SGLT2 inhibitors offer kidney and cardiovascular protection and are generally well tolerated in patients with CKD in whom treatment is indicated, including those with advanced CKD before initiation of kidney replacement therapy. Evidence for their efficacy in patients receiving maintenance dialysis remains limited. Treatment may cause an initial minor, recoverable drop in eGFR and is associated with an increased risk of genital mycotic infections and volume depletion, as well as a rare risk of SGLT2 inhibitor-associated ketoacidosis, which may occur with only mildly elevated or even normal blood glucose concentrations (euglycemic ketoacidosis). MRAs are an option for additional kidney and cardiovascular protection, in particular for diabetic patients who have CKD, but the main limitation remains an increased risk of hyperkalemia. Another class, glucagon-like peptide-1 receptor agonists (GLP-1 RAs), also show cardiovascular and renal benefits in patients with type 2 diabetes and CKD. Still, these medications are associated with gastrointestinal side effects, the need for injections in most cases, and the high price of treatment, which are obstacles to their general use.

Over the past decade, the gut microbiota has emerged as a key regulator of host metabolism, immune homeostasis, and overall health [5,6]. The loss or alteration of a healthy gut bacterial ecosystem, often referred to as dysbiosis, has been linked to the onset and worsening of many chronic conditions, including obesity, type 2 diabetes mellitus, heart disease, inflammatory bowel disease, and CKD [6,7]. The relationship between CKD and the intestinal microbiota is increasingly recognized as bidirectional and is collectively referred to as the gut–kidney axis [8,9]. Progressive loss of kidney function profoundly alters the intestinal microenvironment through accumulation of uremic solutes, metabolic acidosis, dietary restrictions, delayed intestinal transit, intestinal wall edema, and extensive polypharmacy [9,10]. Protein-bound toxins such as indoxyl sulfate (IS), p-cresyl sulfate (PCS), and p-cresyl glucuronide (PCG) increase [8,11,12]. Although numerous studies have characterized microbiome alterations in CKD, substantial heterogeneity persists in specific microbial signatures, their functional consequences, and their relationship to disease severity [13,14]. Variations in CKD etiology, disease stage, dietary habits, medication use, sample size, follow-up duration, sequencing methods, bioinformatics pipelines, and analytical approaches have made it difficult to replicate taxonomic findings across studies [13,14,15].

The intestinal microbiota is a potentially modifiable therapeutic target [16,17]. Many factors affect microbiota composition and blood toxin levels, including uremic toxins, short-chain fatty acids (SCFAs), and inflammatory biomarkers. However, clear evidence of these interventions’ effects on kidney disease, cardiovascular complications, kidney failure, or death remains limited. This discrepancy represents a central unresolved problem in gut–kidney axis research. Rather than offering another descriptive catalog of CKD-associated microbial alterations, this review critically examines the translational gap between biological plausibility, changes in intermediate biomarkers, and clinically meaningful outcomes. Throughout this review, therapeutic evidence is therefore considered across three levels: (1) mechanistic or biological effects, (2) target engagement and intermediate or surrogate outcomes, including changes in microbiota composition, microbial metabolites, inflammatory markers, intestinal permeability, albuminuria, and kidney function measures, and (3) clinically meaningful outcomes, including sustained kidney function decline, kidney failure, cardiovascular events, hospitalization, and mortality. Improvement at one level does not establish benefit at a subsequent level. We evaluate potential explanations at the level of microbial function, host physiology, CKD-related confounding, intervention design, methodological heterogeneity, and endpoint selection, and consider how functional multiomics and patient stratification could improve the development of microbiome-directed therapies.

## 2. Methodology

We identified the literature informing this narrative review by searching PubMed/MEDLINE and Web of Science, covering publications available up to 6 September 2026. The search strategy combined terms related to chronic kidney disease (“chronic kidney disease”, “CKD”) with terms addressing the gut microbiome and gut kidney axis (“gut microbiota”, “gut microbiome”, “gut kidney axis”, “dysbiosis”) and specific mechanistic or therapeutic topics, including “uremic toxins”, “short chain fatty acids”, “metagenomics”, “metabolomics”, “prebiotics”, “probiotics”, “synbiotics”, “postbiotics”, “fecal microbiota transplantation”, “diet”, and “exercise”. We combined terms within each conceptual domain using OR, and combined domains using AND where appropriate. We also screened reference lists of relevant original studies, reviews, and meta-analyses to identify pertinent publications.

We included English-language original research studies, intervention studies, clinical trials, systematic reviews, meta-analyses, and experimental research articles on the relationship between the intestinal microbiome, microbial metabolites, the intestinal barrier, CKD pathogenesis, or microbiome-specific therapies. The articles selected for this review were prioritized based on (a) relevance to primary themes of this review; (b) methodological robustness; (c) relevance to clinical practice; and (d) their contribution to mechanistic understanding or therapeutic translation. Because this was a narrative rather than a systematic review, we did not use a formal risk-of-bias or methodological quality-assessment tool. We conducted a descriptive assessment of methodological strength, considering characteristics particular to the study design, such as study design, sample size, suitability of comparator groups, adjustment for possible confounders, and (for intervention studies) randomization and the clinical importance of the outcomes reported. These considerations informed the weighting and interpretation of individual studies rather than serving as formal eligibility criteria.

In searches with a broad literature base, we prioritized human studies, randomized controlled trials, systematic reviews, and meta-analyses. We also retained experimental studies when they provided important mechanistic context or addressed questions with limited human evidence. Most included articles examined the association between microbiome-associated metabolites or inflammation and kidney or cardiovascular outcomes. Because study populations, CKD stages, microbiome analysis techniques, interventions, and reported outcomes varied, we integrated the evidence narratively.

This review aimed to critically appraise the current evidence and the translation gap between microbiome-related biological effects and clinically meaningful outcomes, rather than provide a comprehensive systematic review of every published paper. Accordingly, we did not identify and select studies according to a predefined systematic-review protocol, and we did not perform formal duplicate screening, quantitative risk-of-bias assessment, or PRISMA-based study selection. The narrative approach necessarily introduces author judgment in study selection and evidence weighting and may therefore be susceptible to selection bias. To mitigate this limitation, we prioritized higher-level human evidence where available, explicitly distinguished experimental findings from human observational and interventional evidence, and interpreted heterogeneous or conflicting findings cautiously.

## 3. The Gut–Kidney Axis

### 3.1. Physiological Basis of the Gut–Kidney Axis

The gastrointestinal tract and kidneys are functionally connected, helping regulate metabolism and coordinate biochemical processes, including nutrient metabolism, immune function, hormone release, and waste product removal. However, new insights confirm the role of the renal system in cooperation with the intestinal bacterial population, forming the gut–kidney axis, a two-way interaction between the intestinal microbiota and the kidney that may affect the body’s overall regulation [8,11].

The nondigestible dietary fiber that reaches the large intestine is broken down into nutrients by saccharolytic bacteria [18,19]. However, microbial metabolism generates precursors of several protein-bound uremic toxins, including indole and p-cresol, which undergo host metabolism before appearing in the circulation as compounds such as IS and PCS [20,21]. Normally, the excretion of these substances occurs mainly through the nephron portion of the kidney. These molecules are transported via organic anion transporters (OAT1 and OAT3), breast cancer resistance protein (BCRP), and multidrug resistance-associated protein 4 (MRP4) on the surface of proximal convoluted tubular cells. Thus, normal kidney function prevents their systemic accumulation and toxicity. However, during CKD, impaired renal clearance and tubular secretion promote the systemic retention of microbiota-derived metabolites, while CKD-associated dysbiosis may alter microbial precursor generation, together contributing to kidney injury and systemic inflammation [10,11] (Figure 1).

### 3.2. CKD-Induced Intestinal Dysbiosis

CKD profoundly alters the intestinal ecosystem, resulting in structural and functional changes that collectively promote microbial dysbiosis [8,11]. Multiple factors contribute to CKD-associated dysbiosis. Progressive urea accumulation is among the earliest metabolic disturbances affecting the intestinal environment. Elevated circulating urea readily diffuses into the gastrointestinal lumen, where bacterial ureases hydrolyze urea into ammonia and ammonium hydroxide [10,22]. At the same time, ammonia directly damages intestinal epithelial cells, altering tight junction protein function and increasing gut permeability [23]. The intestinal environment in CKD is further altered by delayed gastrointestinal transit, a common consequence of autonomic dysfunction, reduced physical activity, dietary modifications, and the widespread use of medications such as phosphate binders, iron supplements, opioids, and potassium-binding resins [12,24]. Slow colonic transit prolongs the residence time of undigested proteins in the distal colon, creating conditions that favor proteolytic bacteria over saccharolytic microbes. This shift in microbial metabolism increases amino acid degradation and turnover, producing kidney-toxic substances such as indole, p-cresol, phenol, hydrogen sulfide, and ammonia [25].

Dietary restrictions imposed on patients with advanced CKD may further exacerbate these microbial alterations. Although clinicians commonly advise limiting dietary potassium to prevent hyperkalemia, this practice often leads to lower intakes of whole grains, fruits, vegetables, and legumes, which are major sources of fermentable fiber [26]. Reduced fiber availability limits substrate for saccharolytic bacteria, resulting in less microbial SCFA production and favoring the expansion of proteolytic microorganisms, which use amino acids as their primary energy source [27]. Similar alterations may occur in patients receiving low-protein diets, where inadequate energy intake increases endogenous protein catabolism and further modifies substrate availability within the intestinal lumen [28].

Beyond metabolite changes, CKD is marked by low-grade, persistent systemic inflammation and immune dysfunction. These processes depend on microbiota composition and function. Disruption of host-microbe homeostasis causes further release of lipopolysaccharides (LPS), peptidoglycans, bacterial DNA, and similar components, which are capable of binding to and activating pattern-recognition receptors, namely, Toll-like receptors (TLRs) and nucleotide-binding leucine-rich repeat receptors (NLRs), as well as triggering various nuclear factor kappa B (NF-κB) signaling pathways that are downstream of TLR/NLR pathways [29,30]. Although numerous microbiome studies have attempted to define a characteristic microbial signature of CKD, the literature remains highly heterogeneous [14,31]. Despite this heterogeneity, several microbial alterations have been consistently reported across CKD populations. Table 1 summarizes the principal changes in gut microbial composition and their functional consequences.

At the phylum level, most studies report a relative expansion of *Proteobacteria* and, to a lesser extent, *Verrucomicrobiota*, whereas changes in *Firmicutes* and *Bacteroidota* are less consistent because these phyla encompass bacterial taxa with markedly different metabolic capacities [15,34]. At lower taxonomic levels, reduced abundance of genera such as *Faecalibacterium*, *Roseburia*, *Anaerostipes*, *Coprococcus*, and *Butyricicoccus*, as well as members of the families Ruminococcaceae, and Lachnospiraceae, has been reported in patients with CKD [15]. In contrast, enrichment of bacteria with urease, uricase, indole-producing tryptophanase, and p-cresol-generating enzymes has been repeatedly demonstrated in advanced CKD [10,35]. Genera such as Enterococcus, Escherichia, Klebsiella, Proteus, Clostridium (selected species), and Enterobacter exhibit enhanced proteolytic activity and substantially contribute to the generation of protein-bound uremic toxin precursors [8]. Changes in microbial diversity in CKD remain inconsistent across studies. A number of studies show a reduction in alpha diversity, or diversity of different microbial species present, as CKD progresses, which can imply reduction in the diversity of the microbial species or even loss of balance among them [15,36], whereas others have found that alpha diversity is relatively stable despite marked differences in bacterial community composition and functional profiles [10,37]. Several factors may explain these discrepancies. Factors that could play different roles include CKD stage and cause, dialysis status, diet, medications, geography, sample handling, sequencing and analysis methods, bioinformatic pipelines, and the diversity indices used. Alpha diversity measures only biodiversity within a sample and does not directly estimate microbial metabolic functions. The same level of alpha diversity can coexist with considerable changes in microbial genes and metabolic systems. Functional analysis complements these results well. Metagenomic and metabolomic analyses report enrichment of pathways involved in urease activity, amino acid fermentation, and the generation of uremic toxin precursors, while pathways related to complex carbohydrate metabolism and SCFA production are reduced [38,39]. Functional analysis could reveal CKD-related changes in microbial communities that taxonomic diversity obscures. However, they do not establish that functional reprogramming is a primary cause of CKD or that functional signatures are more predictive of CKD progression than taxonomic composition. Whether functional microbial profiles are more reproducible across populations and more closely associated with clinically meaningful kidney outcomes remains an open research question. Longitudinal studies integrating standardized metagenomic, metatranscriptomic, and metabolomic analyses are required to distinguish microbial changes that contribute to CKD progression from those that arise as consequences of declining kidney function.

### 3.3. Microbial Metabolites Linking the Gut and Kidney

Intestinal microbiota influence kidney function in several ways, but the primary mechanism is through microbial metabolites rather than the microorganisms themselves. Microbe-derived compounds, or metabolites, likely serve as a primary way gut microbiota interact with their host at the molecular level because they can alter epithelial barrier health, immune function, oxidative stress, and host cellular metabolism. Most of the time, bacterial processes help maintain homeostasis by producing SCFAs, which benefit human metabolism. In CKD, circulating concentrations of several protein-bound uremic toxins increase. Thus, the balance between protective and harmful microbial metabolites is a major regulator of gut–kidney axis function [8,11,12]. Figure 2 illustrates the intestinal origin, host metabolism, renal handling, and major biological pathways of representative microbiota-derived metabolites in CKD, while Table 2 summarizes their origin, experimental evidence, human evidence, and clinical interpretation.

#### 3.3.1. Short-Chain Fatty Acids: Protective Mediators of the Gut–Kidney Axis

SCFAs are the primary products of bacterial fermentation by saccharolytic bacteria, such as *Faecalibacterium prausnitzii*, *Roseburia* spp., *Eubacterium rectale*, and *Anaerostipes* spp., as well as members of the *Ruminococcaceae* and *Lachnospiraceae* families, from the indigestible fraction of dietary fibers [18,51]. These metabolites account for 90–95% of total colonic SCFA production and, beyond the gastrointestinal tract, influence virtually all other physiological systems of the body. Among SCFAs, butyrate plays the greatest role because it is the preferred energy source for colonocytes, helping preserve the gut lining and reduce intestinal “leakiness” [6]. In addition, deacetylase enzymes and SCFAs activate and inhibit specific receptors, prompting immune cells to adjust their actions, such as decreasing inflammatory signal secretion and increasing regulatory T cell (Treg) production. There is evidence that SCFAs can down-regulate inflammation-related pathways (e.g., the NF-κB transcription factor pathway) and, at the same time, increase the production of cytokines with anti-inflammatory properties, like interleukin (IL)-10 and transforming growth factor-beta (TGF-β), while the secretion of cytokines such as tumor necrosis factor-alpha (TNF-α), IL-1, and IL-6 is limited [52,53,54].

Experimental data confirm SCFA’s direct renoprotective effects. Acetate or butyrate administration markedly reduced renal inflammation, oxidative stress, mitochondrial dysfunction, and tubulointerstitial fibrosis in mouse ischemia–reperfusion and diabetic nephropathy models [55,56]. The most commonly suggested mechanisms by which SCFAs confer their positive effects are that they first inhibit the generation of reactive oxygen species (ROS) by quenching nicotinamide adenine dinucleotide phosphate (NADPH) oxidase (NOX) superoxide radicals and other radicals. Second, the TGF-β/Smad pathway, which is mainly fibrogenic, is switched off. Third, adenosine monophosphate-activated protein kinase (AMPK), an important enzyme regulating cellular energy levels, is stimulated. Fourthly, macrophages are reprogrammed through a polarization shift toward anti-inflammatory phenotypes [55]. These findings support the biological plausibility of renoprotective effects of SCFAs. Human studies, however, have mainly documented associations between CKD, reduced abundance of SCFA-producing taxa, and altered SCFA concentrations [32,60]. Whether restoring SCFA production slows CKD progression or improves cardiovascular outcomes remains unclear.

#### 3.3.2. Protein-Bound Uremic Toxins: Central Mediators of CKD Progression

Circulating concentrations of protein-bound uremic toxins (PBUTs) increase markedly as kidney function declines. Importantly, their circulating concentrations reflect the combined effects of intestinal microbial precursor generation and absorption, host metabolism, and renal elimination, including tubular secretion. CKD-associated dysbiosis may increase precursor generation in some settings, but elevated circulating concentrations should not by themselves be interpreted as evidence of increased microbial production. Tryptophan, tyrosine, and phenylalanine are first broken down by intestinal microbes in the colon, which produce indole, p-cresol, and phenol as metabolic byproducts. The liver then absorbs and converts these molecules into IS, PCS, and PCG, respectively [8,35]. Their strong protein binding further limits removal by conventional dialysis [20].

IS is the most extensively studied of these compounds. Bacterial tryptophanase converts dietary tryptophan into indole, which undergoes hydroxylations and sulfations in the liver to produce IS [42]. IS penetrates renal tubular epithelial cells via OAT1 and OAT3. At these sites, it activates the aryl hydrocarbon receptor (AhR), stimulates ROS production, and induces the expression of pro-fibrotic mediators, including TGF-β, connective tissue growth factor (CTGF), and plasminogen activator inhibitor-1 (PAI-1) [43,44]. These pathways drive epithelial-to-mesenchymal transition (EMT), extracellular matrix (ECM) deposition, and chronic tubulointerstitial fibrosis. In addition, IS triggers NF-κB pathways and increases monocyte chemoattractant protein-1 (MCP-1) expression, ultimately worsening the renal inflammatory response [40,61].

PCS is derived from microbial fermentation of tyrosine and phenylalanine and has been implicated in several inflammatory, vascular, and renal effects. Increased levels of PCS in the blood have been associated with endothelial dysfunction, vascular calcification, oxidative stress, cardiovascular events, and all-cause mortality in patients with CKD [45,46]. Mechanistically, PCS increases endothelial ROS production through NADPH oxidase activation, upregulates inflammatory and adhesion molecules, and promotes monocyte-endothelial interactions [62]. Importantly, the interaction between oxidative stress and NO signaling may further contribute to vascular dysfunction in CKD, as altered redox conditions can shift NO signaling from vasoprotective pathways toward reactive nitrogen species formation and endothelial injury [63]. Experimental models also show that PCS accelerates renal fibrosis and activates inflammatory proximal tubular epithelial cells [47].

PCG, unlike PCS, was previously regarded as metabolically inert, which made it a less interesting subject. However, recent research indicates that PCG has an independent pro-inflammatory effect and can induce endothelial dysfunction, likely by stimulating leukocyte-endothelial contacts and oxidative stress pathways [48]. Despite its uncertain role, recently published metabolomic findings show that PCG may be an important factor responsible for CKD-related systemic toxicity [64].

#### 3.3.3. Additional Microbiota-Derived Metabolites

In addition to SCFAs/PBUTs, various other bacterial metabolites play a role in gut–kidney signaling. One example is trimethylamine N-oxide (TMAO), a hepatic oxidation byproduct of dietary choline, phosphatidylcholine, and carnitine, linked to cardiovascular problems in CKD [57]. In addition, elevated TMAO levels have been associated with faster kidney function decline and increased mortality in human studies, while experimental studies suggest a direct role in renal tubulointerstitial fibrosis and dysfunction [65]. In vitro and animal studies reveal that TMAO activates the NLR family pyrin domain-containing 3 (NLRP3)-related inflammatory signaling, increases oxidative stress, and promotes renal fibroblast activation and collagen production, thereby providing potential mechanistic links between gut microbial metabolism, inflammation, and renal fibrosis [66,67].

Other metabolites, including IAA, phenylacetylglutamine (PAGln), HA, secondary bile acids, and hydrogen sulfide, also influence host physiology, though their effects appear more context-dependent [39]. IAA, another tryptophan-derived metabolite, activates AhR signaling and has been linked to endothelial dysfunction and cardiovascular complications in CKD [68]. PAGln has recently attracted considerable interest for its ability to enhance platelet responsiveness via adrenergic receptor signaling, thereby increasing thrombotic risk [69]. Conversely, secondary bile acids regulate the farnesoid X receptor (FXR) and Takeda G protein-coupled receptor 5 (TGR5), which are involved in glucose metabolism, inflammation, and renal fibrosis, underscoring the complexity of microbial-host metabolic interactions [70].

### 3.4. Intestinal Barrier Dysfunction and Systemic Inflammation

#### 3.4.1. Microbial Translocation and Immune Activation

Epithelial barrier deterioration can allow bacterial components and microbial metabolites to migrate from the intestines into the rest of the body. LPS, an outer membrane component of most Gram-negative bacteria, is one of the best-characterized factors responsible for systemic inflammation in CKD [71]. Elevated LPS levels have been repeatedly found in patients with severe CKD and have been linked to inflammatory biomarkers, cardiovascular problems, and death [72]. Recognition of LPS by TLR4 on macrophages, dendritic cells, endothelial cells, and renal tubular epithelial cells triggers the MyD88-dependent signaling pathway, leading to nuclear translocation of NF-κB and transcription of pro-inflammatory cytokines, including TNF-α, IL-1β, IL-6, and monocyte chemoattractant protein 1 (MCP-1) [73]. Persistent activation of these pathways contributes to chronic low-grade inflammation, a hallmark of CKD that is strongly associated with accelerated disease progression and increased cardiovascular risk [74].

LPS is not the only factor that initiates innate immune activation. Bacterial DNA, peptidoglycans, flagellin, and other pathogen-associated molecular patterns (PAMPs) also activate TLRs and NLRs, including the NLRP3 inflammasome [75]. The NLRP3 inflammasome triggers cleavage of pro-caspase-1 into caspase-1, which in turn converts pro-IL-1β and pro-IL-18 into mature forms, activating inflammatory cells and responses [76]. Experimental blockade of the NLRP3 pathway has reduced chronic renal inflammation and kidney fibrosis in kidney disease, underscoring the importance of the gut–kidney axis [77]. Figure 3 summarizes the principal mechanisms linking CKD-associated intestinal barrier dysfunction, microbial translocation, and activation of inflammatory pathways.

#### 3.4.2. Chronic Inflammation as a Driver of CKD Progression

The inflammatory response triggered by intestinal barrier failure does not remain confined to the gastrointestinal tract. It also affects the entire body. Chronic exposure to microbial products will activate the endothelium by inducing higher expression levels of vascular cell adhesion molecule-1 (VCAM-1), intercellular adhesion molecule-1 (ICAM-1), and E-selectin. These molecules, in turn, help recruit leukocytes and cause vascular inflammation [78]. These developments can further lead to endothelial dysfunction, arterial stiffness, and early atherosclerosis [79].

The mechanisms of microbial metabolites and barrier dysfunction overlap rather than being separate. PBUTs, including IS and PCS, not only cause oxidative damage and fibrosis in the kidneys but also disrupt epithelial barriers, allowing microbial passage and promoting persistent inflammation [80]. However, the lack of SCFAs impairs epithelial lining integrity and weakens immunoregulatory functions, paving the way for an enduring pro-inflammatory state. These combined factors ultimately lead to a vicious cycle characterized by dysbiosis, impaired barrier function, systemic inflammation, and kidney failure. A systems-level immunometabolic framework may further help integrate metabolic dysfunction, inflammatory signaling, and mitochondrial stress when interpreting the systemic consequences of gut-derived metabolic disturbances and their potential contribution to disease heterogeneity and differential therapeutic responses [81].

## 4. Microbiome-Targeted Therapeutic Strategies

Because microbiome composition, metabolite handling, dietary exposures, and treatment-related factors differ substantially across the CKD spectrum, evidence from nondialysis CKD, dialysis populations, kidney transplant recipients, specific CKD phenotypes, and experimental models is identified separately where relevant and is not assumed to be directly generalizable across these settings. Recognizing intestinal barrier breakdown as an essential part of kidney disease pathophysiology can yield significant therapeutic benefits. Several microbiome-directed interventions, including dietary fiber, selected prebiotic and probiotic preparations, and microbiota-derived metabolites, have modified intestinal barrier or inflammatory phenotypes in experimental kidney disease. However, the strength and consistency of evidence differ substantially among intervention classes [16,80]. Throughout this review, we describe evidence by source and translational relevance rather than assigning a formal evidence grade. Accordingly, we distinguish (i) mechanistic and preclinical evidence derived from in vitro and animal studies; (ii) observational evidence from human studies; (iii) interventional evidence derived from modifications in microbiome composition, microbial metabolites, inflammatory mediators, or other intermediate outcomes; and (iv) evidence for clinically meaningful outcomes, such as changes in renal function, progression to kidney failure, cardiovascular events, hospitalization, and death. These categories constitute a descriptive framework for interpreting translational maturity and should not be interpreted as a formally graded evidence hierarchy. Where relevant, human evidence is further characterized according to CKD stage, dialysis status, and specific CKD phenotype to avoid extrapolation across clinically distinct populations.

### 4.1. Dietary Interventions

Among microbiome-targeted therapies, dietary changes make the most physiological and clinical sense [12,26]. Various microbiome-targeted therapeutic interventions have distinct yet interacting effects along the gut–kidney axis, ranging from dietary substrate availability and microbial composition to the intestinal barrier and immunity, and to host metabolism (Figure 4).

#### 4.1.1. Dietary Fiber as the Principal Modulator of Microbial Metabolism

Among nutrients, fermentable dietary fiber appears to be the strongest factor shaping the composition and activity of gut organisms [27]. Observational studies consistently report inverse associations between dietary fiber intake and circulating PBUT concentrations, inflammatory biomarkers, and all-cause mortality among patients with CKD [82]. Meta-analyses likewise show modest but significant reductions in serum urea, C-reactive protein (CRP), and IS concentrations with fiber supplementation, although effects on eGFR remain inconsistent [83]. The discrepancy between improvements in inflammatory biomarkers and the lack of consistent effects on kidney function likely reflects the relatively short duration of most intervention studies, limited sample sizes, and the slow progression of CKD. Larger trials with longer follow-up and clinically meaningful renal endpoints are therefore required before dietary interventions can be considered disease-modifying therapies.

#### 4.1.2. Protein Intake and Proteolytic Fermentation

Because CKD is characterized by prolonged intestinal transit, reduced fiber intake, and increased luminal urea availability, proteolytic metabolism becomes progressively favored as kidney function declines [24]. Low-protein diets (LPDs), typically providing 0.6–0.8 g protein/kg/day, have been proposed to reduce substrate availability for microbial toxin production and to decrease renal nitrogen load [28]. Very-low-protein diets (VLPDs), supplying approximately 0.3–0.4 g protein/kg/day and supplemented with ketoanalogues of essential amino acids, have demonstrated additional reductions in serum urea concentrations and delayed dialysis initiation in selected patients with advanced CKD [84]. Beyond these nephrological benefits, reduced dietary protein availability may decrease microbial production of indole and p-cresol, thereby lowering circulating IS and PCS concentrations [28].

Nevertheless, excessive protein restriction remains controversial. Inadequate protein intake may contribute to protein-energy wasting (PEW), sarcopenia, frailty, and increased mortality, particularly among older adults and patients receiving dialysis [85]. Moreover, reducing protein intake alone does not necessarily restore microbial eubiosis if dietary fiber intake remains insufficient. Current evidence therefore supports a balanced nutritional approach that emphasizes replacing animal protein with fiber-rich, plant-derived protein sources rather than indiscriminate protein restriction [86].

#### 4.1.3. Plant-Based and Mediterranean Dietary Patterns

Plant-based and Mediterranean diets are rich in vegetables, fruits, legumes, whole grains, nuts, and olive oil, and they involve a low intake of red and processed meat [87]. These dietary patterns supply the microbiome with plenty of fermentable fibers, polyphenols, unsaturated fatty acids, and antioxidant compounds, resulting in a more diverse microbial ecosystem, higher SCFA production, and lower proteolytic fermentation activity. These effects support better maintenance of the intestinal barrier, suppression of protein-bound toxic compound formation during uremia, and a likely anti-inflammatory role at the systemic level, making these approaches appealing for CKD patients. Several observational studies have found a link between adherence to Mediterranean diets and a decline in various inflammatory conditions, a reduced risk of heart disease, a slower progression of kidney function loss, and fewer deaths in people with CKD [88,89]. Mainly plant-based diets yield similar positive results. This dietary pattern results in less acidic foods, a richer source of beneficial bacterial metabolites, and lower production of PBUT precursors derived from aromatic amino acids [90]. Furthermore, replacing animal proteins with plant proteins appears to lower circulating concentrations of IS and PCS while preserving adequate nutritional status with careful supervision [91].

Adopting plant-based dietary strategies for patients with CKD is difficult because most fiber-rich foods are also high in potassium. As hyperkalemia is a potentially fatal complication of advanced CKD, patients with advanced CKD are advised to avoid fruits, vegetables, legumes, and whole grains. Ironically, these restrictions reduce fermentable fiber intake and could worsen gut dysbiosis [86]. Tailored potassium management, combined use of potassium-binding drugs, and proper vegetable preparation could help patients on a nephrological diet eat plant products safely [92]. This system change highlights the challenge of preserving kidney function while maintaining microbial health.

#### 4.1.4. Current Evidence and Future Perspectives

Although dietary interventions consistently benefit microbial metabolism and inflammatory biomarkers, many shortcomings remain. Randomized clinical trials (RCTs) often have small sample sizes, varied diets, short intervention durations, and substantial differences in microbiome sequencing methods. As a result, clinical outcomes such as CKD progression, cardiac events, dialysis initiation, or mortality have been very sparsely studied [93].

### 4.2. Prebiotics

Prebiotics are selectively utilized substrates metabolized by beneficial intestinal microorganisms that promote host health. In patients with CKD, research has primarily focused on fermentable dietary fibers, including inulin, fructooligosaccharides (FOS), galactooligosaccharides (GOS), resistant starch, β-glucans, arabinoxylans, and pectin-derived oligosaccharides [94].

#### 4.2.1. Mechanisms of Action

Under normal conditions, fermentable carbohydrates reach the colon largely undigested, where they serve as substrates for saccharolytic bacteria such as *Bifidobacterium*, *Lactobacillus*, *Faecalibacterium prausnitzii*, and *Roseburia*. By increasing substrate availability for saccharolysis, prebiotics simultaneously reduce bacterial reliance on fermentation products for amino acids [95]. The switch from proteolytic to saccharolytic metabolism is the main mechanism by which prebiotics modulate the gut–kidney axis [96].

#### 4.2.2. Experimental Evidence

Preclinical studies show that selected prebiotic substrates can modify microbial metabolism and several experimental features of kidney disease, although effects vary by substrate and disease model. In adenine-induced CKD rats, oligofructose-enriched inulin reduced circulating PCS and modified inflammatory and oxidative stress-related parameters, although it did not significantly reduce IS [97]. In the Cy/+ rat model of progressive CKD, dietary inulin altered microbial community composition and reduced circulating gut-derived uremic toxins. These changes were accompanied by improvements in several manifestations of CKD mineral and bone disorder, including vascular and cardiac calcification and cardiac fibrosis, although kidney function itself was not improved [98].

More recent experimental studies have provided additional mechanistic evidence. In gut humanized CKD mice, oat resistant starch and oat β-glucan increased SCFA concentrations, reduced IS and PCS, improved intestinal barrier integrity, and attenuated renal inflammation and fibrosis through pathways involving NF-κB and TGF-β [99]. In experimental diabetic kidney disease (DKD), inulin type fructans increased SCFA-producing bacteria and acetate concentrations and reduced glomerular injury and renal fibrosis. Antibiotic depletion and fecal microbiota transfer experiments supported a microbiota-dependent component of these effects [100].

These studies strengthen the biological plausibility that fermentable prebiotic substrates can influence kidney-related phenotypes by altering microbial metabolism, SCFA production, intestinal barrier function, and uremic toxin generation. However, the heterogeneity of prebiotic compounds, experimental models, and reported outcomes precludes treating these effects as a uniform class effect. Recent systematic evidence likewise indicates variable microbiome responses to different dietary fibers in CKD, emphasizing the need to distinguish individual substrates rather than generalizing findings across all prebiotics.

#### 4.2.3. Clinical Evidence in CKD

Clinical trials of prebiotic supplementation in CKD have produced heterogeneous results, with effects depending on the substrate, population, dose, and outcome assessed. In a randomized crossover trial of 39 patients with CKD, Poesen et al. found that four weeks of arabinoxylan oligosaccharide supplementation did not significantly reduce serum PCS, PCG, IS, or phenylacetylglutamine, although they observed a small reduction in TMAO [101].

In a double blind, placebo-controlled randomized trial involving 50 patients with nondialysis CKD and an eGFR below 45 mL/min/1.73 m^2^, Ramos et al. evaluated FOS supplementation at 12 g/day for three months. FOS produced a nonsignificant trend toward lower total and free serum PCS but did not significantly affect IS, IAA, eGFR, intestinal permeability markers, or most inflammatory outcomes [102]. A subsequent analysis of this intervention found reduced IL-6 but no significant improvement in arterial stiffness or overall endothelial function [103].

More recently, researchers have evaluated resistant starch in patients with moderate CKD. In a double-blind RCT involving 68 patients with CKD stages G3a to G4, 16 weeks of high-amylose resistant starch supplementation reduced PCS and altered selected features of the gut microbiota, providing evidence that resistant starch can modify microbial metabolism in nondialysis CKD [104].

These individual trials should nevertheless be interpreted cautiously. A meta-analysis of 14 RCTs involving 513 participants found that microbiota-directed interventions collectively reduced circulating PCS but did not significantly reduce IS. Subgroup analyses suggested a reduction in PCS with prebiotics, although the included interventions and patient populations were heterogeneous [105]. More specifically, a recent meta-analysis of 10 resistant starch trials comprising 355 participants reported small decreases in IS and blood urea nitrogen (BUN), without significant changes in PCS, IL-6, TNF-α, albumin, or phosphorus [106].

#### 4.2.4. Limitations and Future Perspectives

Prebiotics are generally safe and well tolerated, but at higher doses, gastrointestinal side effects such as bloating, abdominal discomfort, flatulence, and diarrhea may reduce treatment compliance. In addition, significant differences in microbiome composition among individuals are an important factor to consider. In such cases, the same prebiotic may elicit markedly different metabolic responses, depending on the subject’s baseline microbial environment, diet, medications, and CKD stage [94]. Emerging evidence further suggests that next-generation prebiotics could target microbial pathways involved in SCFA production and PBUT generation. Future studies integrating metagenomic, metabolomic, and dietary data may help identify patients most likely to respond to specific prebiotic interventions.

### 4.3. Probiotics

Probiotics are live microorganisms that, when administered in adequate amounts, confer a health benefit on the host [107]. In CKD, they are among the most extensively studied microbiome-targeted therapies and are intended to restore microbial homeostasis by increasing beneficial bacterial populations, suppressing urease- and protease-producing microorganisms, and reducing gut-derived uremic toxin production [8].

#### 4.3.1. Mechanisms of Action

Probiotics exert their beneficial effects through diverse, synergistic mechanisms. They compete with pathogenic microorganisms for nutrients and adhesion sites, which not only hinders pathogen growth but also produces lethal substances such as bacteriocins, lactic acid, hydrogen peroxide, and other acids. These substances inhibit the activity of certain bacteria that produce urease and protease, for instance, members of the *Enterobacteriaceae*. Probiotics also strengthen the intestinal barrier by modulating tight junction protein levels and promoting goblet cell mucus production, leading to a less leaky gut and reducing the risk of bacteria entering the bloodstream through gaps in the gut barrier [107]. Probiotics also influence the host immune system by activating regulatory T-cells and boosting anti-inflammatory cytokine synthesis, while suppressing inflammatory signals by blocking NF-κB pathway activation. Overall, these mechanisms promote sugar fermentation, increase SCFA production, minimize protein-bound uremic toxins, and reduce overall inflammation [8,108,109].

#### 4.3.2. Experimental Evidence

Preclinical evidence suggests that selected probiotic strains can modify gut-derived toxin exposure and renal injury in animal models of CKD, but the effects are strain- and model-specific. In a 5/6 nephrectomy mouse model, Lactobacillus rhamnosus L34 administered for 14 weeks reduced renal fibrosis, serum creatinine, proteinuria, circulating IS, TMAO, endotoxin, and TNF-α compared with untreated nephrectomized controls. The study also showed anti-inflammatory effects of L34 conditioned medium in intestinal and tubular cell models, supporting a strain-specific mechanism beyond changes in microbial abundance alone [110].

A more recent study evaluated *Bifidobacterium longum* subsp. *longum* BL21 in a rat model of CKD. Six weeks of supplementation altered gut microbial composition and significantly reduced circulating IS, IAA, and trimethylamine N-oxide, with smaller effects on PCS and PCG. These findings support a direct effect of this strain on microbial metabolite profiles, but the study did not establish a generalized renoprotective effect applicable to other probiotic strains [111].

In a 5/6 nephrectomy rat model, *Bifidobacterium animalis* subsp. *lactis* Bi 07 reduced bacterial translocation, intestinal permeability, circulating endotoxin, and inflammatory biomarkers after four weeks of treatment. This study primarily supports effects on intestinal barrier dysfunction and systemic inflammation rather than hard renal outcomes [112].

#### 4.3.3. Clinical Evidence

Clinical trials of probiotics in CKD have produced heterogeneous results, with effects depending on the probiotic formulation, study population, and outcome assessed. In a randomized, double-blind, placebo-controlled trial in patients with nondialysis CKD, Borges et al. assigned 46 patients receiving maintenance hemodialysis to a probiotic formulation containing *Streptococcus thermophilus*, *Lactobacillus acidophilus*, and *Bifidobacterium longum* at 90 billion colony-forming units per day or placebo for three months. Probiotic supplementation did not reduce circulating IS, PCS, IAA, CRP, or IL-6 compared with placebo and did not significantly modify the assessed gut microbiota profile. These findings provided no evidence of improvement in the principal uremic toxin or inflammatory outcomes evaluated in this population [113].

In patients receiving maintenance hemodialysis, Lim et al. evaluated a high-dose multistrain probiotic preparation, including *Lactococcus lactis*, *Lactobacillus salivarius*, and *Lactobacillus pentosus*. They conducted a placebo-controlled, double-blind, randomized trial in hemodialysis patients. The probiotic supplement reduced serum IS levels over six months compared with the placebo group. However, the study found no significant between-group differences in PCS, blood urea nitrogen, inflammatory markers, or markers of microbial translocation. Thus, the biochemical effect was largely restricted to IS rather than reflecting a broad improvement across uremic and inflammatory biomarkers [114].

More recently, Jang et al. conducted a multicenter, randomized, double blind, placebo-controlled study of *Lactobacillus acidophilus* KBL409 at 1 × 10^10^ colony-forming units per day. The per-protocol analysis included 34 participants who received KBL409 and 30 who received placebo. After 16 weeks, serum IS was significantly lower in the KBL409 group than in the placebo group, accompanied by changes in several bacterial genera. This trial provides additional evidence that selected probiotic strains can modify circulating IS and gut microbial composition, but it does not establish that these changes translate into slower CKD progression or improved patient-centered outcomes [115].

The heterogeneity of these individual trials is consistent with meta-analytic evidence. Differences in probiotic strains, doses, treatment duration, CKD stage, dialysis status, background diet, and outcome assessment limit comparisons across studies [105].

#### 4.3.4. Why Are Clinical Results Inconsistent?

Differences between promising experimental data and relatively modest clinical outcomes likely arise from several methodological and biological factors. First, different probiotic products can vary in bacterial species and strain combinations, vitality, microbe count, formulation type, and treatment duration. Probiotic efficacy is generally determined by the strains of bacteria, which means that the results of one form do not reflect a generic effect of that class [107]. However, advanced CKD is marked by severe bacterial imbalance and loss of ecological diversity, leading, at least to some extent, to a gut environment that, if resistant to colonization, is difficult for ingested or injected microorganisms to colonize. Most probiotics on the market survive only briefly in the gastrointestinal tract, and few colonize the host’s gut after intake has stopped [8,116]. Therefore, continued administration or coadministration with substrates that promote probiotic colonization may be required for an ongoing therapeutic effect.

Most importantly, the extent to which a new dietary habit influences probiotic efficacy is significant. The effects of probiotic strains may depend on the surrounding microbial and nutritional environment. This has contributed to interest in synbiotic approaches that combine live microorganisms with selectively utilized substrates [117,118].

Finally, the major problem in most studies is the use of relatively small numbers of patients, short treatment periods of only several weeks or even months, and the inclusion of patients at different CKD stages. These constraints greatly reduce statistical power, making it difficult to identify clinically relevant outcomes. Another important limitation is that many commercially available probiotic strains were originally selected for gastrointestinal disorders rather than CKD-specific metabolic functions. Consequently, successful intestinal colonization does not necessarily translate into reduced uremic toxin production or improved renal outcomes, highlighting the need for next-generation probiotics with targeted metabolic properties.

#### 4.3.5. Safety Considerations

Probiotic interventions have generally been well tolerated in the relatively small CKD trials conducted to date, with gastrointestinal symptoms among the most commonly reported adverse effects. However, available evidence is insufficient to establish safety across all CKD populations. The most commonly reported side effects are mild gastrointestinal symptoms, including bloating and flatulence [119]. Still, caution is required when probiotics are prescribed to heavily immunocompromised individuals, critically ill patients, or those who require central venous catheters, as these patients are prone to infection. Even a rare but possible case of bacterial or fungal infection can be a concern [120]. Although such complications are uncommon, proper selection of the patient group for a probiotic drug is paramount.

#### 4.3.6. Future Perspectives

Future strategies may include next-generation probiotics, such as *Faecalibacterium prausnitzii* and *Akkermansia muciniphila*, as well as engineered strains designed to modify uremic toxin precursor metabolism or deliver anti-inflammatory products [121,122]. Their efficacy and safety in CKD require clinical evaluation.

### 4.4. Synbiotics

Synbiotics combine probiotics and prebiotics in a single treatment to harness their complementary effects through distinct mechanisms of action. In CKD, this dual approach has attracted significant attention because severe microbial dysbiosis can limit the effectiveness of probiotics when used alone [8,117]. The International Scientific Association for Probiotics and Prebiotics (ISAPP) first defined synbiotics as “combinations containing both living microorganisms and non-digestible substrates which are to be selectively consumed only by microorganisms in the gut that are helpful to human health”. The latest agreement on synbiotics differentiates two types of synbiotics: “complementary” synbiotics (probiotic-prebiotic pairs whose effects are separate but can have a joint positive effect), and “synergistic” synbiotics (where the prebiotic is chosen to support the growth and metabolic activities of the co-administered probiotic) [117,123]. This distinction is particularly relevant for CKD, as most commercially available products fall into the complementary category, whereas true synergistic formulations remain relatively uncommon.

#### 4.4.1. Biological Rationale

The theoretical advantage of synbiotic therapy lies in its ability to overcome several limitations of isolated probiotic supplementation. Colonization by administered probiotic strains is often transient because the dysbiotic intestinal environment characteristic of CKD provides limited ecological support for their persistence [8,116]. Reduced availability of fermentable carbohydrates, increased luminal urea concentrations, delayed intestinal transit, and expansion of proteolytic bacteria create conditions unfavorable for sustained probiotic activity [8,80]. Prebiotic supplementation may partially overcome these limitations by supplying fermentable substrates that selectively stimulate both administered probiotics and endogenous saccharolytic microorganisms. The therapeutic rationale is that synbiotics simultaneously restore saccharolytic metabolism and reduce PBUT production.

#### 4.4.2. Experimental Evidence

Preclinical studies show that selected synbiotic formulations can modify gut microbial composition and metabolite production and attenuate some features of experimental CKD, although the evidence remains formulation- and model-specific. In an adenine-induced CKD rat model, Yang et al. showed that synbiotic treatment reduced the abundance of indole-producing *Clostridium* and fecal indole concentrations and partially restored microbial diversity compared with untreated CKD animals [124]. These findings provide experimental evidence that synbiotic treatment can modify microbial composition and intestinal generation of a uremic toxin precursor.

Jerez Morales et al. evaluated a synbiotic combining *Lactobacillus bulgaricus* 6c3 with inulin and FOS in a 5/6 nephrectomy rat model. After 16 weeks, serum IS increased significantly in untreated nephrectomized animals but remained essentially unchanged in synbiotic-treated animals. Renal fibrosis progressed less in the synbiotic group, whereas cardiac fibrosis was not reduced and serum creatinine did not differ significantly between treated and untreated nephrectomized animals [125].

More recently, a synbiotic containing *Bifidobacterium longum* BFS309, *Lactobacillus salivarius* LBR228, inulin, and chitosan oligosaccharide was evaluated in a cisplatin-induced CKD rat model with dietary phosphate loading. After 12 weeks, synbiotic-treated animals had lower serum phosphate, calcium phosphate product, parathyroid hormone (PTH), and IS concentrations than untreated CKD animals [126]. These findings extend the experimental evidence for synbiotics to CKD-associated mineral and bone abnormalities, although confirmation in other experimental models is required.

Overall, these experimental studies support the biological activity of selected synbiotic formulations, but findings should be interpreted cautiously and not generalized across formulations or experimental models. First, synbiotics differ widely in bacterial species, prebiotic substrates, formulation amounts, exposure duration, and CKD models used in experiments. Few studies compare a synbiotic product with its separate components, such as the probiotic and prebiotic parts. As a result, current preclinical evidence cannot be generalized to determine whether synbiotics are better than probiotics or prebiotics taken separately. Moreover, the effects observed with one synbiotic formulation cannot necessarily be generalized to other formulations within this category.

#### 4.4.3. Clinical Evidence

SYNERGY, the best reported clinical trial of synbiotic therapy in patients with nondialysis CKD, was a crossover placebo-controlled, double-blind, randomized trial by Rossi et al. Thirty-seven nondialysis CKD patients with an eGFR between 10 and 30 mL/min/1.73 m^2^ were randomly assigned to receive either six weeks of synbiotic treatment or a placebo, with 31 patients completing both regimens. Synbiotic treatment did not appreciably decrease serum IS, but serum PCS fell by 14 µmol/L, and the stool microbiome pattern shifted, with *Bifidobacterium* increasing and *Ruminococcaceae* decreasing. Kidney function did not change, and albuminuria mildly increased during the synbiotic treatment session. This suggests that changes in microbial composition and toxin levels do not translate into measurable renal benefits [118].

In a randomized, placebo-controlled, double-blind trial of synbiotic supplementation in 34 patients with nondialysis CKD and an eGFR of 15 to 45 mL/min/1.73 m^2^, *Lactobacillus acidophilus*, *Lactobacillus casei*, and *Bifidobacterium lactis* + inulin were administered for 12 weeks, and synbiotic therapy gave a greater reduction in serum IS than the placebo group. However, the between-group difference in PCS was not statistically significant, and synbiotic supplementation did not significantly improve eGFR or albuminuria [127].

In patients receiving maintenance dialysis who had constipation, Lydia et al. conducted a double blind RCT of *Lactobacillus acidophilus* and *Bifidobacterium longum* for 60 days. The intervention primarily assessed IS, constipation symptoms, and constipation-related quality of life. This trial shows that synbiotic effects may differ by dialysis status, baseline gastrointestinal phenotype, and formulation, and reinforces the need to avoid extrapolating results from one CKD population to another [128].

In the SYNERGY II feasibility trial, 68 adults with nondialysis stage 3–4 CKD were randomized to synbiotic therapy or placebo for 12 months. Synbiotic treatment altered the stool microbiome, including enrichment of *Bifidobacterium* and *Blautia*, but did not improve circulating IS, PCS, or other major exploratory outcomes. Unexpectedly, eGFR decreased by approximately 3.1 mL/min/1.73 m^2^ and serum creatinine increased in the synbiotic group, findings that require cautious interpretation but argue against assuming that favorable microbiome changes necessarily translate into kidney protection [119].

Meta-analytic evidence reflects this heterogeneous picture. A systematic review and meta-analysis of RCTs found that synbiotic interventions were associated with a modest reduction in circulating PCS but not IS. The available trials were small, used diverse probiotic strains and prebiotic substrates, and were generally too short to evaluate kidney failure, cardiovascular events, hospitalization, or mortality [105].

#### 4.4.4. Why Have Synbiotics Not Fulfilled Their Therapeutic Promise?

Several biological and methodological factors may explain the discrepancy between encouraging preclinical findings and the modest effects observed in clinical trials. One factor that could account for differences in synbiotic effects between research settings and clinical situations is the high degree of individual variation in microbial dysbiosis. Patient factors such as microbial composition, metabolic capacity, dietary habits, medication use, disease severity, and more may all play a part [6]. Consequently, the biological response to a given synbiotic formulation may vary substantially between individuals. Second, many available synbiotic formulations have been developed empirically rather than through a mechanism-based selection of complementary probiotic strains and prebiotic substrates. Many formulations combine commonly used probiotic strains with prebiotic substrates without establishing whether the selected substrate preferentially supports the administered microorganisms. This practice severely limits the potential for real synergistic associations and may explain why many clinical investigations show only small metabolic benefits [116]. Finally, rebuilding a normal microbial community may not be enough to correct the severe metabolic defects that accompany advanced CKD. A uremic state with high levels of urea in the intestinal lumen, inflammation, acidosis, slow bowel movements, and frequent antibiotic use still imposes selective pressure on microbes that can break down proteins [8,80]. Consequently, microbiome-targeted interventions may require integration with dietary modification and optimized CKD management to achieve clinically meaningful effects.

#### 4.4.5. Future Perspectives

Future synbiotic development should shift from empirically combined products to mechanism-based formulations in which probiotic strains and prebiotic substrates are selected based on complementary metabolic functions. Integration of metagenomic and metabolomic profiling may facilitate this approach, but its clinical advantage over conventional formulations remains to be established [117,121].

### 4.5. Postbiotics

Postbiotics are preparations of inanimate microorganisms and/or their components that confer a health benefit on the host. According to the ISAPP consensus definition, a postbiotic must contain inactivated microbial cells or cellular components, although the preparation may also contain microbial metabolites. Purified microbial metabolites, such as isolated SCFAs, bile acids, peptides, or other microbial products, should not themselves be classified as postbiotics. These compounds are therefore considered separately here as microbiota-derived metabolites or metabolite-based therapeutic approaches [129].

#### 4.5.1. Mechanisms of Action

The biological activity of postbiotic preparations may arise from several microbial structures retained after inactivation, including cell wall components, surface proteins, pili, lipoproteins, and microbial nucleic acids. Depending on the microorganism and preparation method, these components can interact with pattern recognition receptors expressed by intestinal epithelial and immune cells, including TLRs and lectin receptors. Such interactions may influence epithelial barrier function, innate and adaptive immune responses, and inflammatory signaling. Metabolites retained within a postbiotic preparation may additionally contribute to its biological activity, but their presence alone does not define a preparation as a postbiotic. Consequently, the biological effects of a specific postbiotic cannot necessarily be extrapolated to other preparations because microbial strain, growth conditions, matrix, inactivation procedure, and final composition may all influence biological activity [129].

#### 4.5.2. Experimental Evidence

Experimental evidence specifically evaluating postbiotics in kidney disease remains limited. Studies in other experimental settings indicate that preparations containing inactivated microorganisms or their cellular components can modify epithelial barrier function and immune signaling. Potential mechanisms include modulating pattern recognition receptor signaling, enhancing epithelial barrier integrity, and regulating inflammatory responses [129]. However, these effects depend heavily on the microbial strain and preparation method.

Importantly, experimental studies involving purified butyrate, acetate, propionate, or other isolated microbial metabolites should not be interpreted as evidence for postbiotic therapy. Although experimental data provide a rationale for investigating preparations of inactivated microorganisms in CKD, direct evidence demonstrating renoprotective effects of appropriately defined postbiotics remains insufficient.

#### 4.5.3. Clinical Evidence

Clinical evidence for postbiotic preparations in patients with CKD, including both nondialysis and dialysis populations, is currently very limited. Most human studies examining microbiome-directed changes in CKD have assessed dietary changes and prebiotic, probiotic, or synbiotic interventions rather than formulations of deceased microorganisms as defined by the current ISAPP guidelines [129]. Because of this, it remains largely unknown if postbiotic preparations can enhance kidney function, reduce albuminuria, slow progression to kidney failure, or improve cardiovascular or mortality outcomes in CKD patients.

Similarly, studies involving direct administration of purified microbial metabolites represent metabolite-based interventions rather than postbiotic interventions. This distinction matters when evaluating translational evidence because mechanistic effects of individual microbial metabolites cannot establish the clinical efficacy of preparations containing inactivated microorganisms [129].

Accordingly, postbiotics should currently be considered an experimental microbiome-directed strategy in CKD. Well-designed clinical studies using clearly characterized preparations that satisfy consensus definitions are required before their potential therapeutic value can be established.

#### 4.5.4. Advantages over Live Microorganism-Based Therapies

Postbiotic preparations have several theoretical advantages over interventions containing viable microorganisms. Because their activity does not require survival or colonization of administered microorganisms, their effects may be less dependent on whether an administered strain can persist within the dysbiotic intestinal environment of advanced CKD. The absence of a requirement for microbial viability may also facilitate manufacturing, storage, formulation, and standardization [129].

Another potential advantage concerns safety. Because appropriately manufactured postbiotics are completely devoid of live microorganisms, the likelihood of complications from microbial reproduction, bacterial spread, and infection should, in theory, be lower than with therapies based on live microorganisms. This advantage may apply mostly to individuals with compromised protective mechanisms, such as those undergoing immunosuppressive treatment for their conditions. Nevertheless, the absence of viable microorganisms does not establish clinical safety. The safety of individual preparations must be demonstrated in the intended population, and microbial composition, inactivation procedures, residual components, and potential contaminants require appropriate characterization [129].

These potential advantages do not extend to purified microbial metabolites. Although such compounds can also be standardized and administered without viable microorganisms, they constitute a separate therapeutic category and should be evaluated according to their individual pharmacological and biological properties [129].

#### 4.5.5. Limitations and Future Perspectives

The principal limitation of postbiotic research in CKD is the scarcity of studies evaluating preparations that meet the current consensus definition. Heterogeneous terminology and inclusion of purified metabolites, secreted products, or cell-free preparations in the historical literature further limit interpretation and comparison across studies [129].

Future CKD studies should report the identity of the progenitor microorganism or microorganisms, growth conditions, inactivation method, composition of the final preparation, and evidence confirming the absence of viable organisms. The biological activity and safety of the final preparation should then be demonstrated in the intended target population. Particular attention should be given to distinguishing changes in microbial or inflammatory biomarkers from clinically meaningful outcomes.

At present, there is insufficient evidence to consider postbiotics an established treatment for CKD. Their main value lies in providing a potentially standardized way to exploit beneficial microbial structures and associated bioactive components without administering viable microorganisms. Whether this theoretical advantage translates into improvements in kidney failure, cardiovascular events, hospitalization, or mortality requires evaluation in appropriately designed clinical trials.

### 4.6. Microbiota-Derived Metabolites as Therapeutic Agents

Microbiota-derived metabolites represent a therapeutic concept distinct from postbiotics. According to the ISAPP consensus definition, substantially purified microbial metabolites are not classified as postbiotics without microbial cellular biomass [129]. Thus, purified SCFAs, bile acid derivatives, peptides, and other isolated microbial products should be considered according to their individual biological and pharmacological properties rather than grouped under the term postbiotic. This distinction is particularly relevant in CKD, where several microbial metabolites have strong mechanistic links with intestinal barrier function, inflammation, immune regulation, and kidney injury, but evidence supporting their direct therapeutic administration remains predominantly preclinical [130,131].

#### 4.6.1. Short-Chain Fatty Acids

SCFAs, mainly acetate, propionate, and butyrate, are products of intestinal microbial fermentation of nondigestible carbohydrates and represent one of the best-understood functional aspects of the intestinal microbiota’s influence on host physiology. They also serve as a nutrient source for colonocytes, mainly through butyrate uptake. SCFAs also serve as energy substrates for colonocytes, with butyrate representing their primary energy source. SCFAs affect host signaling through G protein-coupled receptors (GPRs) and inhibition of histone deacetylases (HDACs). These pathways modulate epithelial barrier integrity, immune cell differentiation, inflammatory signaling, and cellular metabolism [18,132].

Experimental studies provide evidence for direct biological effects of SCFAs in specific animal models of kidney disease. Corte Iglesias et al. [133] found that propionate and butyrate attenuated inflammatory and profibrotic responses in tubular cells and reduced renal injury in a mouse model of the transition from acute kidney injury (AKI) to CKD. Importantly, these effects were demonstrated after direct SCFA administration, supporting biological activity independent of changes in microbial composition. In contrast, human evidence remains predominantly observational. In patients with CKD, fecal propionate and butyrate concentrations decreased across more advanced CKD stages and were associated with kidney function measures. However, these findings were observational and do not demonstrate that SCFA deficiency causes CKD progression or that supplementation improves clinical outcomes.

Liu et al. [134] provided additional experimental evidence, showing that acetate, propionate, and particularly butyrate attenuated kidney injury in a rat model of anti-glomerular basement membrane glomerulonephritis. Butyrate therapy decreased urine protein, serum creatinine, crescent formation, complement activation, and renal infiltration of inflammatory cells. The treatment was also linked to changes in T cell responses. Overall, these results provide mechanistic support for possible renoprotective effects of SCFAs, although they remain to be confirmed experimentally.

In observational human CKD cohorts, abnormal SCFA levels and a loss of SCFA-producing microbes in patients with CKD, but SCFA levels are strongly influenced by the stage of kidney disease, dietary habits, dialysis/transplant status, and the type of bio-sample tested [32]. Thus, experimental studies support biological activity of SCFAs in selected kidney disease models, whereas human evidence remains predominantly observational. Whether direct SCFA administration improves kidney or cardiovascular outcomes in CKD remains unknown.

#### 4.6.2. Other Microbial Metabolites

The therapeutic relevance of microbiota-derived metabolites extends beyond SCFAs. Intestinal microorganisms generate or modify numerous bioactive compounds, including indole derivatives, secondary bile acids, TMA-derived metabolites, polyamines, and other small molecules that interact with host receptors and metabolic pathways [135,136]. Consider these metabolites individually because their biological effects, pharmacokinetics, target receptors, and potential toxicity differ substantially.

Tryptophan metabolism provides a particularly important example. Intestinal microorganisms convert tryptophan into several indole derivatives that can interact with host receptors, particularly the AhR and pregnane X receptor (PXR), and thereby influence epithelial barrier integrity, mucosal immunity, oxidative stress, and inflammatory signaling [130,137]. However, microbial tryptophan metabolism can generate compounds with markedly different biological effects. The host absorbs indole and subsequently converts it to IS, which accumulates as kidney function declines and has been implicated in oxidative stress, inflammation, fibrosis, and cardiovascular toxicity.

Indole-3-propionic acid illustrates the context-dependent nature of these metabolites. Experimental work in DKD has suggested protective effects of indole-3-propionic acid on glomerular endothelial mitochondrial function, accompanied by lower circulating concentrations in patients with DKD [138]. In contrast, recent experimental work in cisplatin-induced CKD found that indole-3-propionic acid administration aggravated renal inflammation and fibrosis through AhR- and NF-κB-related signaling [139]. These apparently divergent findings emphasize that a microbial metabolite should not be classified as universally beneficial or harmful without considering disease context, concentration, tissue exposure, and downstream receptor signaling.

Microbial transformation of bile acids represents another potentially important pathway. Intestinal microorganisms deconjugate and modify primary bile acids to generate secondary bile acids, thereby altering signaling through receptors including FXR and Takeda G protein-coupled receptor 5 (TGR5). These pathways participate in lipid and glucose metabolism, mitochondrial function, inflammation, and immune regulation [140]. Human microbiome and metabolome analyses have demonstrated alterations in microbial functions related to secondary bile acid metabolism in advanced kidney disease [141]. However, evidence that direct manipulation or administration of individual microbiota-derived bile acid species improves CKD outcomes remains insufficient.

Other microbial products, including peptides and extracellular molecules, may exert immunomodulatory or barrier-protective effects. However, purified peptides, proteins, exopolysaccharides, or other isolated microbial products should not automatically be classified as postbiotics [129]. Their therapeutic potential requires compound-specific characterization, including pharmacokinetics, dose–response relationships, target engagement, safety, and evidence of clinical efficacy.

#### 4.6.3. Translational Limitations

Several barriers separate the mechanistic promise of microbiota-derived metabolites from their clinical application in CKD. First, circulating metabolite concentrations reflect the combined effects of microbial generation, intestinal absorption, host metabolism, tissue distribution, and renal elimination. This issue is particularly important in CKD because declining glomerular filtration and impaired tubular secretion can substantially alter systemic metabolite concentrations independently of microbial production. Consequently, an association between a circulating microbial metabolite and eGFR cannot establish either increased microbial generation or a causal contribution to CKD progression [130,141].

Second, microbiome composition and circulating metabolite concentrations are not interchangeable measures of microbial function. In a human study combining microbiome and metabolome profiling, Wang et al. [141] demonstrated relationships among microbial functions, fecal metabolites, circulating uremic metabolites, and renal failure phenotypes, but these relationships remained dependent on both microbial metabolism and host handling. Similarly, Wu et al. [32] demonstrated that dietary protein restriction was associated with changes in microbial composition and serum metabolites in CKD, while IS and PCS concentrations did not differ between dietary groups. These observations illustrate why changes in microbial taxa should not automatically be interpreted as corresponding changes in systemic toxin exposure.

Third, the biological effects of individual metabolites depend on concentration, tissue, receptor, and disease context [142]. Manipulating a single metabolite may therefore fail to reproduce the coordinated metabolic output of a healthy microbial ecosystem. The contrasting experimental effects reported for indole-3-propionic acid in different kidney disease models further illustrate this problem [138,139]. Moreover, metabolites generated within the intestinal lumen may have local biological effects that differ from those resulting from systemic pharmacological administration.

Finally, most therapeutic evidence remains derived from cell culture and animal models. Future studies should combine metagenomic and metabolomic characterization with direct measurement of microbial metabolite generation, where feasible, and rigorous assessment of host clearance. Intervention studies should establish pharmacokinetics, dose–response relationships, safety, and target engagement before evaluating clinically meaningful efficacy. Until such evidence becomes available, purified microbiota-derived metabolites should be considered experimental therapeutic candidates rather than established microbiome-directed treatments for CKD.

### 4.7. Oral Adsorbents

Unlike interventions targeting diet or the microbiome to restore microbial balance, oral adsorbents act against a main downstream effect of gut dysbiosis: excess uremic toxins of gut origin. Oral adsorbents aim to reduce intestinal uptake of toxin precursors before they enter the bloodstream [143]. Among currently available agents, AST-120 (Kremezin^®^, Kureha Corporation, Tokyo, Japan) is the oral adsorbent that has been tested most thoroughly. AST-120 consists of porous spherical activated carbon particles that remain in the gastrointestinal lumen and adsorb low-molecular-weight organic compounds, mostly indole and other bacterial metabolites, products of tryptophan fermentation [144]. Because indole is the precursor to IS, reducing its intestinal absorption also lowers hepatic conversion to IS, ultimately decreasing circulating toxins. AST-120 differs from traditional charcoal products. It is engineered to maximize adsorption capacity while minimizing systemic absorption and exhibits minimal gastrointestinal toxicity.

#### 4.7.1. Mechanisms of Action

The rationale for using AST-120 as a therapeutic agent is to prevent systemic exposure to gut toxins, rather than to change bacterial species composition. Bacteria degrade dietary tryptophan, producing indole, which is absorbed across the intestinal epithelium and transported to the liver. The liver then oxidizes and sulfatizes it, forming IS. Similarly, bacteria produce p-cresol when they metabolize tyrosine and phenylalanine, and it is converted into PCS and PCG [8,143]. With reduced kidney function, these toxic waste products accumulate because decreased tubular secretion via OAT1 and OAT3 hampers their removal. As a result, systemic toxicity increases gradually. AST-120 acts as a physical barrier at the intestinal mucosal level, binding indole and other related aromatic compounds in the gastrointestinal tract before absorption. These effects include reduced activation of the oxidative stress pathway, reduced TGF signaling, reduced AhR activation, reduced inflammation, and restored endothelial function, all of which the literature links to CKD development and progression [143,144,145,146,147]. Potentially, the major mechanism of AST-120’s therapeutic activity is adsorbing microbial toxins that cause systemic damage, thereby reducing host exposure. As a result, rather than microbiome-modifying therapies, oral adsorbents such as AST-120 should be considered microbiome-metabolite modulators.

#### 4.7.2. Experimental Evidence

Preclinical studies have demonstrated effects of AST 120 on renal, vascular, and cardiac abnormalities in experimental CKD. In 5/6 nephrectomized rats, AST 120 reduced circulating IS and attenuated renal oxidative stress, as reflected by lower acrolein and 8-hydroxy-2′deoxyguanosine levels. Treatment was also associated with less glomerulosclerosis and tubulointerstitial fibrosis, supporting a relationship between reduced uremic toxin exposure, oxidative stress, and renal structural injury [148].

Vascular effects have also been demonstrated experimentally. In subtotally nephrectomized rats, Namikoshi et al. found that AST 120 reduced serum IS and improved endothelium-dependent vascular responses, with no significant differences in renal function or renal histology between treated and untreated animals. AST 120 also reduced aortic nitrotyrosine accumulation and p47phox expression, suggesting attenuation of vascular oxidative and nitrative stress [149].

Lekawanvijit et al. examined cardiac effects in 5/6 subtotally nephrectomized rats. AST 120 reduced circulating IS and decreased left ventricular interstitial fibrosis by approximately 68%, together with reductions in cardiac TGF-β and activated NF-κB signaling. These findings provide experimental evidence linking reduction in IS exposure with attenuation of CKD-associated cardiac fibrosis [150].

#### 4.7.3. Clinical Evidence

Early clinical investigations of AST 120 focused largely on biochemical changes and surrogate measures of kidney function rather than hard renal outcomes. By adsorbing intestinal indole, AST 120 reduced circulating IS in some clinical studies of patients with nondialysis CKD, although the magnitude and consistency of this effect varied across studies. Thus, early clinical evidence showed biological target engagement but did not establish that lowering circulating IS slowed CKD progression [151,152].

The multinational EPPIC 1 and EPPIC 2 trials provided the most definitive clinical evaluation. Schulman et al. randomized 2035 adults with moderate to severe CKD to AST 120 at 9 g/day or placebo in addition to standard therapy. The lead composite endpoint was defined as either the start of dialysis therapy, kidney transplant, or serum creatinine level doubling. AST 120 did not significantly extend time to the primary outcome in EPPIC 1 (HR: 1.03, 95% CI: 0.84 to 1.27) or EPPIC 2 (HR: 0.91, 95% CI: 0.74 to 1.12). Overall, both studies were assessed as neutral [145].

A subsequent randomized open-label trial conducted by Cha et al. enrolled 579 patients with nondialysis stage 3 or 4 CKD and compared AST 120 at 6 g/day plus conventional treatment with conventional treatment alone for up to 36 months. The primary composite outcome comprised doubling of serum creatinine, a ≥50% reduction in eGFR, or initiation of kidney replacement therapy. AST 120 did not significantly improve the primary renal outcome. Moreover, although serum and urinary IS concentrations decreased during follow-up, the changes did not differ significantly between treatment groups [151].

Meta-analytic evidence supports this distinction between biochemical and clinical outcomes. A meta-analysis of eight studies involving 3349 participants found that AST 120 significantly reduced serum IS compared with placebo or conventional treatment, with a weighted mean difference of 0.28 mg/dL, but did not significantly reduce the composite renal outcome (RR 0.97, 95% CI 0.88 to 1.07) or all-cause mortality (RR 0.94, 95% CI 0.73 to 1.20) [153]. A subsequent network meta-analysis of 15 RCTs involving 3763 participants suggested potential benefits with tailored dosing for kidney failure and composite renal outcomes, whereas fixed low- and high-dose regimens were not superior to control [154]. Collectively, these findings illustrate that reduction in IS and other surrogate markers does not necessarily translate into improved kidney or survival outcomes.

#### 4.7.4. Why Has AST-120 Shown Limited Clinical Efficacy?

If IS production was completely blocked, many other metabolites, such as PCS, PCG, TMAO, phenylacetylglutamine, and inflammatory microbial products, would remain largely unchanged. Second, restoring healthy gut flora with AST-120 is outside its scope, and it does not repair epithelial barrier dysfunction. As a result, the orally administered adsorbent targets the consequence of dysbiosis rather than the root cause [155].

Several factors, including high blood pressure, diabetes, metabolic acidosis, fibrosis from stress, and inflammation, drive kidney failure. Patient adherence is another practical limitation because AST-120 requires a substantial treatment burden, while gastrointestinal adverse effects may further reduce tolerability [144,145,146]. Several factors, such as hypertension, diabetes mellitus, metabolic acidosis, oxidative stress, fibrosis, and chronic inflammation, each independently contribute to declining kidney function, regardless of the level of microbial toxin buildup. So, if only one pathological pathway is reduced, it may not be sufficient to achieve a measurable, clinically significant improvement in short-term randomized trials [2].

#### 4.7.5. Emerging Toxin-Lowering Strategies

Researchers are investigating novel oral sorbents with improved adsorption specificity and reduced pill burden. Additional strategies include inhibiting bacterial enzymes that produce toxin precursors, selectively modulating microbial amino acid metabolism, developing engineered probiotics that degrade uremic toxin precursors, and implementing precision nutritional interventions to reduce proteolytic fermentation [8,16].

#### 4.7.6. Future Perspectives

Despite a lack of consistent evidence from large-scale randomized trials demonstrating clinically significant renal protection with AST-120, the drug’s development strongly supports the idea that microbial metabolites may represent modifiable therapeutic targets in CKD. Accordingly, new therapeutic strategies are expected to focus increasingly on selectively modulating microbial metabolic pathways, in combination with dietary prebiotics, probiotics, synbiotics, and postbiotics, rather than on adsorbing all components [8,9]. Within this evolving therapeutic landscape, oral adsorbents should be viewed not as stand-alone disease-modifying agents but as components of a broader multimodal strategy to restore metabolic homeostasis within the gut–kidney axis. Identification of patients with particularly high circulating PBUT concentrations, combined with precision metabolomic profiling and individualized treatment selection, may ultimately improve clinical efficacy and facilitate the translation of toxin-lowering therapies into routine nephrological practice [8].

### 4.8. Fecal Microbiota Transplantation

Fecal microbiota transplantation (FMT) represents the most comprehensive microbiota-based therapeutic strategy available today, aiming to restore the intestinal ecosystem by transferring a complex microbial community from a healthy donor to a recipient with gut microbiota dysbiosis. While probiotics, prebiotics, and synbiotics target individual bacterial strains or specific metabolic pathways, FMT aims to replace a disturbed microbial ecosystem with a healthy, diverse microbiome capable of performing a wide array of metabolic functions. Because CKD is accompanied by major changes in microbial composition, decreased biodiversity, impaired SCFA production, and increased PBUT levels, FMT has recently been considered a tool to re-establish microbial balance in the gut and support kidney function [156,157,158]. Clinically, FMT has successfully treated recurrent *Clostridioides difficile* infection after antibiotic treatment, showing that restoring a healthy microbial ecosystem can significantly affect human illness [159]. This success has been a driving force for research on the use of FMT for dysbiotic diseases such as inflammatory bowel disease, metabolic syndrome, liver disease, neurological disorders, and, more recently, CKD. Although FMT in nephrology is not yet implemented in clinical practice, a steadily growing body of preclinical evidence suggests that altering the microbial ecosystem can reduce the underlying pathogenic mechanisms driving CKD.

#### 4.8.1. Biological Rationale

FMT aims to modify dysbiotic microbial communities by restoring microbial diversity, cross-feeding interactions, and ecological resilience. Rather than introducing isolated bacterial strains, transplantation transfers a complete microbial ecosystem, including bacteria, bacteriophages, fungi, archaea, and microbial metabolites, which collectively contribute to intestinal homeostasis [10,160]. Restoration of these ecological interactions may increase SCFA production, improve epithelial barrier integrity, suppress systemic inflammation, and reduce microbial generation of PBUTs.

FMT may also modulate the host’s immune system through mechanisms beyond its metabolic effects. Restoration of a more balanced microbial community has been proposed to promote regulatory T-cell responses, attenuate TLR/NF-κB-mediated inflammatory signaling, and increase anti-inflammatory cytokines such as IL-10. However, these immunomodulatory effects have been demonstrated mainly in experimental models and non-CKD settings, and their contribution to FMT effects in human CKD remains uncertain [161]. Enhanced epithelial barrier function reduces systemic translocation of bacterial endotoxins, thereby reducing chronic immune activation that contributes to CKD development.

#### 4.8.2. Experimental Evidence

Preclinical FMT studies in animal models of CKD provide evidence that intestinal microbial communities can modify selected features of experimental kidney disease, although the direction and magnitude of these effects depend strongly on the donor microbiota, recipient model, and experimental protocol. Importantly, FMT has not consistently improved renal injury across CKD models. In adenine-induced CKD mice, Barba et al. evaluated repeated FMT from healthy control mice. Although transplantation modified intestinal microbial composition and improved glucose tolerance, it did not significantly improve serum creatinine or proteinuria, attenuate renal expression of TGF-β1, IL-6, or TNF-α, or reduce histological fibrosis. These findings show that restoring selected microbiota features does not necessarily translate into improved kidney injury [162].

Stronger evidence for a causal contribution of the microbiota comes from transplantation experiments using CKD and healthy donors. Liu et al. used microbiota from rats with severe CKD induced by 5/6 nephrectomy and from sham-operated controls as donors for microbiota-depleted rats with mild CKD induced by 1/2 nephrectomy. Recipients of CKD-derived microbiota developed greater PBUT accumulation and more severe renal injury, whereas transplantation of microbiota from healthy donors suppressed microbial pathways involved in tryptophan metabolism and lysine degradation and reduced PBUT accumulation. This reciprocal donor design provides stronger experimental evidence that the composition and metabolic function of the intestinal microbiota can modify CKD-related phenotypes rather than merely change secondary to kidney dysfunction [163].

More recently, researchers evaluated FMT in a unilateral ureteral obstruction model of renal fibrosis. FMT increased renal and circulating monocytic myeloid-derived suppressor cells and was associated with lower proinflammatory cytokine expression and attenuated renal fibrosis. Depletion and adoptive transfer experiments further implicated these cells in the observed immunomodulatory and antifibrotic effects, providing mechanistic evidence linking microbiota manipulation with immune regulation during experimental renal fibrosis [164].

#### 4.8.3. Clinical Evidence

Clinical evidence for FMT in CKD remains limited and is derived from one small randomized trial and a small number of observational reports. Given the heterogeneity in CKD stage, dialysis status, underlying kidney disease, and intervention protocols, findings from nondialysis CKD, dialysis populations, and other clinical settings should not be considered directly interchangeable. Arteaga Muller et al. conducted a single-center, double blind, randomized, placebo-controlled trial in 28 patients with CKD stages 2 to 4 secondary to diabetes and/or hypertension. Participants received oral FMT capsules or placebo for six months. FMT was associated with changes in microbial composition, including increased abundance of *Roseburia* spp., and adverse events were limited primarily to mild or moderate gastrointestinal symptoms. Although fewer participants in the FMT group met the investigators’ definition of CKD progression than in the placebo group, serum creatinine, creatinine clearance, and 24 h proteinuria did not differ significantly between groups during follow-up. The small sample size and single-center design therefore preclude conclusions regarding preservation of kidney function [165].

Zhong et al. retrospectively evaluated washed microbiota transplantation (WMT) in patients with kidney dysfunction. 86 patients with kidney dysfunction underwent WMT therapy; most had minor impairment: 76 were CKD G2, nine were G3, and only one was classified as G4. Compared with patients with kidney dysfunction who did not receive WMT, serum creatinine, eGFR, and BUN improved after WMT. WMT also increased microbial diversity and shifted microbial composition toward healthy donor microbiomes in the group with microbiome data. Urine metagenomics showed higher levels of specific metabolites such as HA, cinnamoylglycine, and indole, all linked to gut bacteria. WMT-related adverse events occurred in 2.91% of procedures, and no serious adverse events were reported. However, the retrospective design, predominance of mild kidney dysfunction, gastrointestinal indications for WMT in many participants, and limited microbiome and metabolomic sampling substantially restrict causal interpretation [166].

Additional evidence is limited to small uncontrolled observations. A report describing FMT in a patient with biopsy-proven focal segmental glomerulosclerosis (FSGS) documented stabilization of kidney function and reduced proteinuria following FMT, along with changes in metabolic and inflammatory parameters. Because this observation involved an individual patient receiving concomitant glucocorticoid therapy, it cannot establish that FMT caused the clinical improvement [167].

#### 4.8.4. Challenges and Safety Considerations

Several important limitations currently prevent FMT from becoming a widespread nephrology treatment. First, donor selection is labor-intensive because the gut community includes thousands of microbial species, including viruses, fungi, and bacteriophages. Meticulous donor screening is essential to substantially reduce the risk of transmitting infectious agents, multidrug-resistant pathogens, and undesirable microbial traits [156,157]. According to international guidelines, prospective stool donors should undergo comprehensive clinical assessment and appropriate blood and stool testing before acceptance, with repeat screening or donation-specific testing performed according to the donor program and applicable protocol. Second, individuals respond to treatment in widely varying ways. Engraftment is neither uniform nor guaranteed and depends on multiple factors, including the recipient’s baseline microbiota composition, antibiotic exposure, dietary habits, immune status, and degree of CKD-associated dysbiosis [168]. In addition, donor-derived material is inherently heterogeneous, with substantial interindividual variation in microbial composition and functional capacity. Individual donor strains differ in their ability to colonize and persist in the recipient, with engraftment influenced by both donor and recipient characteristics [168,169]. This variability in donor material and engraftment limits the reproducibility and standardization of FMT and represents an important challenge for its translation beyond established indications. A third difficulty is creating a consistent method of administration (such as colonoscopy, nasogastric tube, or oral capsules). Only recently has it been acknowledged that uremic toxins are strongly linked to gut dysbiosis, suggesting that the gut microbiome following FMT will decay very slowly in CKD patients under persistent uremic conditions. In any case, even though FMT, when followed by standardized protocols, is overall a safe procedure, there have been a few cases where the procedure has resulted in a major adverse incident, such as transmission of multidrug-resistant organisms and infectious outbreaks, so that regulatory bodies have decided to tighten the donor screening requirements [170].

#### 4.8.5. Emerging Alternatives to Conventional Fecal Microbiota Transplantation

Recognition of the limitations of conventional FMT has stimulated the development of next-generation microbiome therapeutics. Defined microbial consortia composed of cultured bacterial strains aim to replicate the therapeutic effects of FMT while avoiding the variability associated with donor-derived stool [171]. Such “live biotherapeutic products” offer greater standardization, quality control, and regulatory oversight. Another promising approach is precision microbiota transplantation, in which donor selection is guided by metagenomic and metabolomic profiling to maximize the restoration of microbial functions deficient in individual patients [161]. Upcoming interventions may involve engineered microbial communities, microbiota-derived next-generation probiotics, bacteriophages, and microbiome therapy, aiming to replicate the metabolic functions of FMT without the logistical and safety issues associated with donor-collected fecal matter [172].

### 4.9. Exercise and Lifestyle Modulation of the Gut Microbiome

In addition to dietary factors, which are mainly regarded as lifestyle strategies for altering the gut microbiota, physical activity and other lifestyle-related changes also affect the microbial profile, metabolic activity, and interactions between the host and its microbiota. Exercise is a standard prescription for CKD patients, mainly because it confers cardiovascular benefits, improved physical function, better insulin sensitivity, and a higher quality of life. However, exercise was only recently shown to significantly affect the gut microbiome, so it is now considered a new lifestyle intervention that may influence the gut–kidney axis [79,173]. Unlike probiotics or FMT, exercise does not directly change microbial composition by supplementing microorganisms. Rather, it indirectly alters the intestinal environment, thereby creating favorable conditions for beneficial gut microbiota [173,174]. These findings suggest that exercise may influence microbial function indirectly through changes in host physiology, although the contribution of microbiome remodeling to the established systemic benefits of exercise remains uncertain.

#### 4.9.1. Mechanisms Linking Exercise and the Gut Microbiome

The human gut can benefit from regular physical activity through multiple interconnected physiological mechanisms. Physical activity shortens intestinal transit time, reducing prolonged exposure to proteins retained in the intestinal lumen that would otherwise undergo proteolytic fermentation and produce nephrotoxic metabolites [173,174]. A faster gastrointestinal transit rate may reduce metabolite generation by gut microbes, such as indole, p-cresol, ammonia, and PBUT precursors. Physical exercise is also linked to better insulin sensitivity, which supports improved glucose metabolism, reduced adipose tissue inflammation, and enhanced glucose disposal in skeletal muscle, thereby preserving systemic metabolic balance. These metabolic adaptations influence nutrient availability in the intestinal lumen, which, in turn, shapes microbial substrate utilization. In addition, physical exercise releases myokines such as irisin and IL-6, which have immunomodulatory effects and, in turn, affect host-microbe interactions [175]. Another key factor is strengthened epithelial barriers. For instance, some studies indicate that even moderate physical activity increases tight junction protein levels, which also enhance mucus production and intestinal blood flow, thereby reducing intestinal leakiness and the transport of bacterial endotoxins such as LPS [174]. These effects could reduce continuous stimulation of TLR-mediated inflammatory pathways and may thereby contribute to lower systemic inflammation. However, this microbiome-mediated pathway has not been demonstrated to modify CKD progression in humans. Lastly, physical exercise increases microbial richness and the proliferation of SCFA-producing microbes, such as *Faecalibacterium*, *Roseburia*, *Akkermansia*, and members of the *Lachnospiraceae* family [176]. Higher SCFA production has been linked experimentally to enhanced epithelial barrier, regulatory T cell differentiation, suppression of NF-κB signaling, and improved mitochondrial function. Whether exercise-induced changes in SCFA production mediate clinical benefits in CKD remains unknown.

#### 4.9.2. Experimental Evidence

Animal studies outside the CKD setting have shown that exercise can alter gut microbial composition, increase the abundance of SCFA-producing bacteria, and improve intestinal barrier integrity. However, direct experimental evidence demonstrating these microbiome-mediated effects in CKD models remains limited [177]. Preliminary research shows that exercise creates an environment in which microorganisms prefer to break down carbohydrates rather than proteins. As a result, the most abundant members of the bacterial community are SCFA producers that ferment carbohydrates more efficiently. These microbial shifts could theoretically reduce proteolytic fermentation and PBUT precursor generation, although this has not been directly demonstrated in CKD [176]. Experimental microbiota-transfer studies suggest that the gut microbiome may contribute to some systemic effects of exercise, as transplantation of microbiota from exercise-trained mice to sedentary recipients can partially reproduce metabolic, vascular, and anti-inflammatory effects observed with exercise [178]. These findings provide experimental evidence that gut microbiota alterations can contribute to selected exercise-associated physiological effects in specific animal models, but do not establish microbiome mediation of exercise benefits in CKD.

#### 4.9.3. Clinical Evidence in CKD

Clinical investigations of exercise-induced microbiome remodeling in CKD are sparse but growing. Regular aerobic and resistance exercise improves physical performance, cardiorespiratory fitness, blood pressure control, and health-related quality of life in patients with CKD, with evidence also supporting benefits for muscular function. Exercise also decreases circulating inflammatory biomarkers, such as CRP, TNF-α, and IL-6, while improving insulin sensitivity and endothelial function [179]. Importantly, these established benefits of exercise in CKD should not be interpreted as evidence that changes in the gut microbiome mediate them.

Exercise increases microbial diversity and the abundance of SCFA-producing taxa in non-CKD individuals. Studies on exercise in CKD mainly focus on the loss of beneficial SCFA-producing species as a consequence of frailty and impaired physical function, rather than directly evaluating physical activity itself [79]. Dietary modifications increase fecal SCFA-producing microbiota and decrease circulating protein-bound uremic toxins in patients with CKD. However, because no combined interventional studies are available, it is unclear whether exercise alone has a significant effect beyond dietary changes [91].

Most human microbiome research has focused on healthy athletes rather than patients with CKD. Consistently, elite athletes exhibit greater microbial diversity and higher fecal concentrations of SCFAs, including acetate, propionate, and butyrate, compared with more sedentary controls [180]. Although these findings cannot be directly extrapolated to CKD, they provide biological plausibility for there being an effect of physical activity on microbiome composition and function.

#### 4.9.4. Lifestyle Factors Beyond Exercise

Other lifestyle and environmental factors, such as sleep and circadian rhythm, psychological stress, cigarette use, drug exposure and other environmental exposures, may also influence gastrointestinal microbial composition and function [181,182,183,184]. These factors can affect microbial diversity, intestinal barrier function, and microbial interactions within the human host. The contribution these factors make to CKD-associated dysbiosis, and whether they could have a role as microbiome-targeted therapies in CKD, has not yet been clearly established, although they are an area of potential interest, more so than currently available therapies except diet and exercise.

#### 4.9.5. Limitations and Future Perspectives

CKD-specific evidence linking exercise-induced microbiome changes to clinical outcomes remains limited, with available studies generally small, observational, and heterogeneous in exercise modality and intensity. Diet, medications, CKD stage, comorbidities, and baseline microbial composition further complicate attribution of microbiome changes specifically to exercise [173,185]. Future studies integrating microbiome profiling with objective measures of physical activity are needed to determine whether microbiome modulation contributes independently to the established benefits of exercise in CKD.

Table 3 summarizes the principal microbiome-targeted therapeutic strategies discussed throughout this review, along with their primary mechanisms of action, current clinical evidence, and major limitations.

## 5. Conclusions

Studies so far strongly suggest that the link between CKD and gut microbiological changes is tight. Microbiological changes include alterations in microbial community composition and microbial-derived metabolite production. They also lead to changes in gut wall permeability and in the levels of several microbiota-related metabolites transported into the bloodstream. Experimental studies suggest that these alterations may promote inflammation, fibrosis, vascular dysfunction, and kidney injury. However, their causal contribution to CKD progression in humans remains uncertain. Human studies have predominantly been observational or have focused on changes in microbiota composition, SCFAs, protein-bound uremic toxins, inflammatory biomarkers, or intestinal permeability. Although microbiome-targeted interventions can modify several of these surrogate measures, robust evidence that such changes translate into slower eGFR decline, delayed kidney failure, fewer cardiovascular events, reduced hospitalization, or lower mortality remains lacking. Interpretation of microbiota-related metabolites also requires careful separation of microbial generation from host metabolism and renal elimination. IS and PCS, which circulate in the blood, indicate not only intestinal precursor levels but also reduced renal clearance and impaired tubular secretion. Because of this, higher levels of such compounds in circulation should not be taken as proof of increased microbial production or of microbiome activity as the cause.

Future research should therefore move beyond descriptive taxonomic associations and short-term biomarker studies. Priorities include standardized microbiome and metabolome methodology, prospective longitudinal studies, direct assessment of microbial metabolite generation where feasible, careful characterization of host clearance, mechanistically informed patient stratification, and randomized trials with clinically meaningful kidney and cardiovascular endpoints. Microbiome-directed therapies should currently be regarded as investigational approaches supported by varying degrees of mechanistic and surrogate evidence rather than established disease-modifying treatments for CKD.

## Figures and Tables

**Figure 1:**
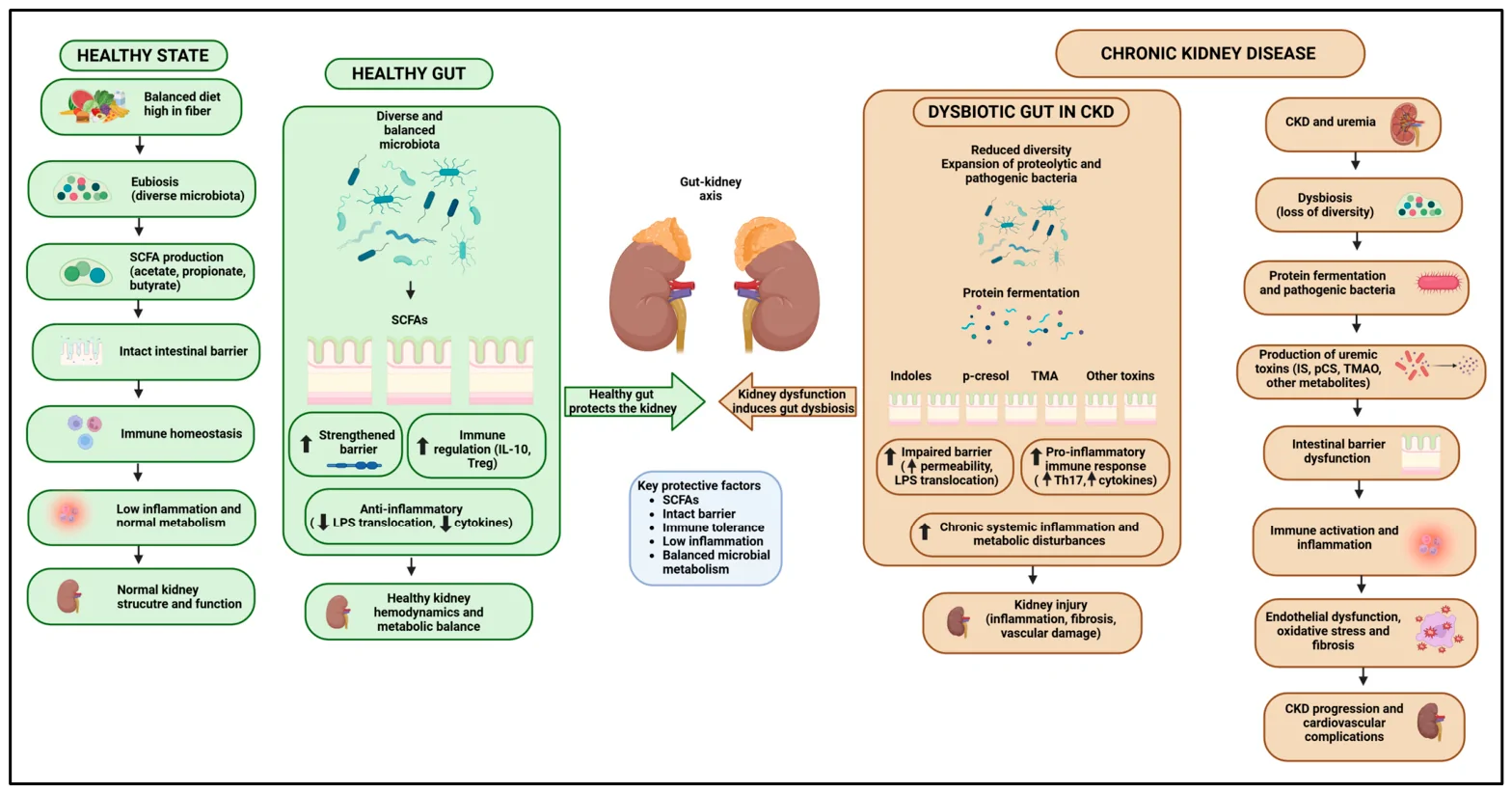
The gut–kidney axis in health and chronic kidney disease (CKD). Diagram depicting two-way communication between the intestinal microbiome and the kidneys. A high-fiber diet promotes healthy intestinal eubiosis, which, through microbial saccharolytic fermentation of fiber, generates short-chain fatty acids (SCFAs). SCFAs help maintain an intact intestinal barrier, support immune balance, and normal kidney function. In CKD, kidney failure can lead to gut bacterial overgrowth and changes in gut bacterial structure. Combined with reduced SCFA production and increased gut permeability, these changes allow microbial products to enter the bloodstream, resulting in systemic inflammation, oxidative stress, vascular changes, and ongoing kidney damage. Declining kidney function may therefore further promote intestinal dysbiosis, contributing to a bidirectional gut–kidney feedback loop. Abbreviations: CKD, chronic kidney disease; SCFAs, short-chain fatty acids; IS, indoxyl sulfate; PCS, p-cresyl sulfate; TMA, trimethylamine; TMAO, trimethylamine N-oxide; LPS, lipopolysaccharide.

**Figure 2:**
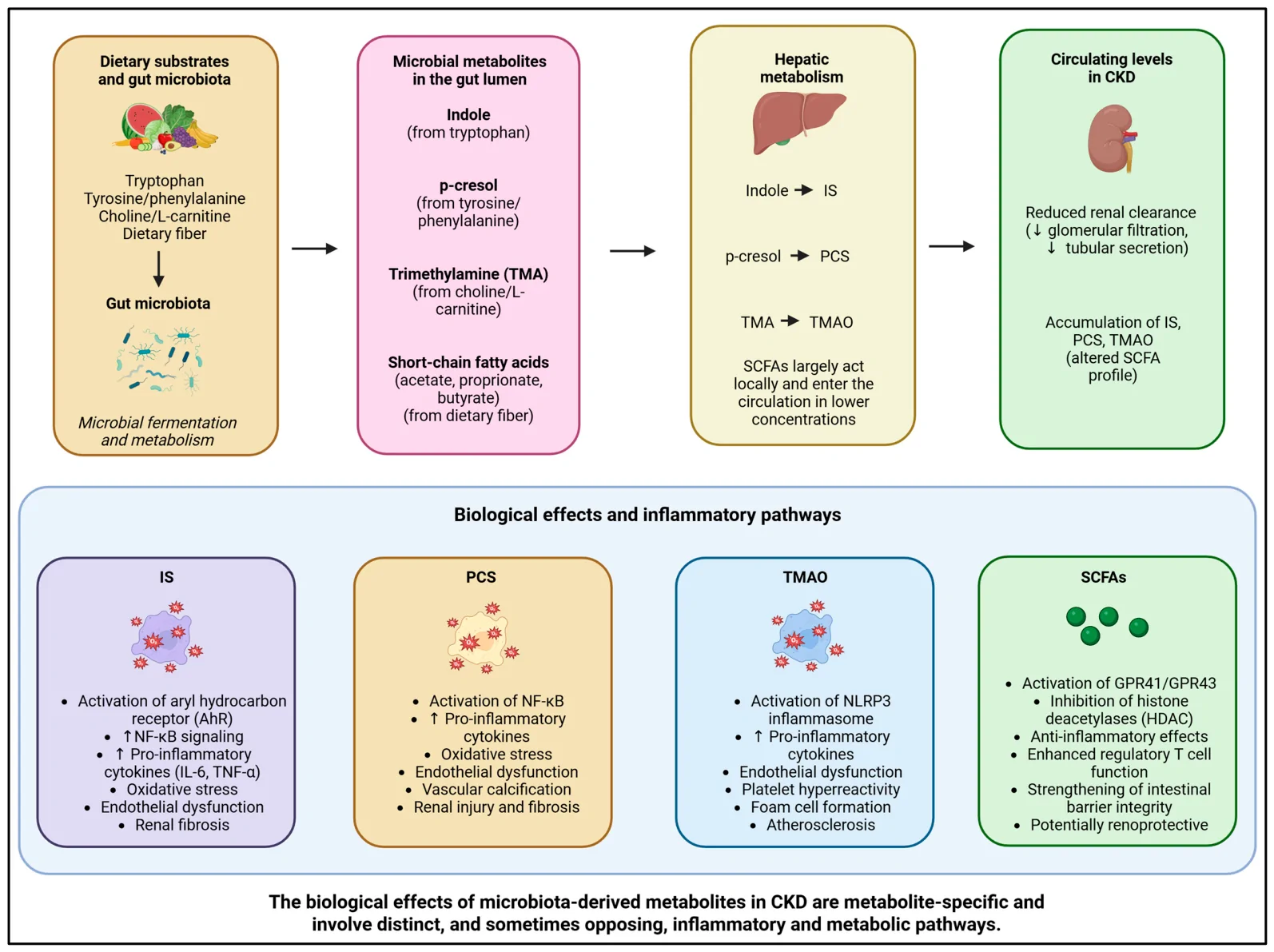
Microbiota-derived metabolites in CKD. Human food substrates are digested into different types, including amino acids, carbohydrates, and lipids. These substrates undergo microbial degradation in the gut, producing microbial metabolites and their precursors, including indole, p-cresol, trimethylamine, and SCFAs. Our body converts microbial indole, p-cresol, and trimethylamine into IS, PCS, and TMAO, respectively. In CKD, decreased glomerular filtration rate and reduced tubular secretion increase levels of several microbiota-related metabolites in the circulatory system. These molecules have specific biological effects, including mechanisms involving AhR, NF-κB activation, oxidative stress, endothelial dysfunction, NLRP3 inflammasome activation, and fibrosis-promoting signaling pathways. However, SCFAs are known to have mostly anti-inflammatory and pro-intestinal barrier functions by binding to G protein-coupled receptors and inhibiting histone deacetylase. Abbreviations: AhR, aryl hydrocarbon receptor; CKD, chronic kidney disease; GPR, G protein-coupled receptor; HDAC, histone deacetylase; IS, indoxyl sulfate; NF-κB, nuclear factor kappa B; NLRP3, NLR family pyrin domain containing 3; PCS, p-cresyl sulfate; SCFAs, short-chain fatty acids; TMA, trimethylamine; TMAO, trimethylamine N-oxide.

**Figure 3:**
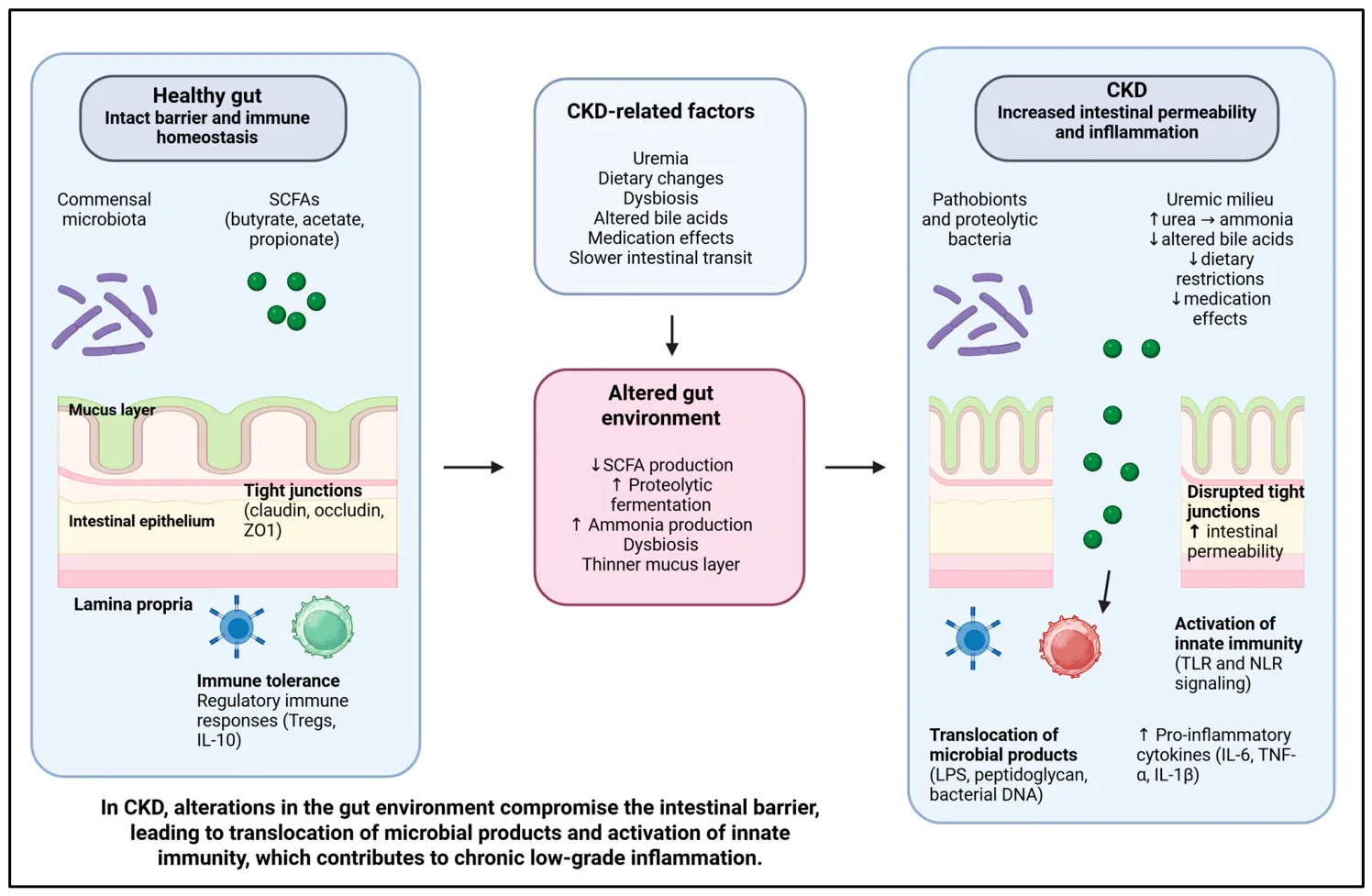
Compromised intestinal barrier integrity and inflammation in CKD. Reduced gut barrier strength and inflammation are commonly observed features in individuals with CKD. CKD, gut urea accumulation, dysbiosis, a shortage of health-promoting microbial metabolites, and other kidney-disease-related factors can alter the gut environment. Increased gut urea and its conversion to ammonia, along with low SCFAs and microbiota imbalance, damage mucus secretions and epithelial tight junctions. Increased intestinal leakiness allows microbial materials such as LPS, peptidoglycan, and bacterial DNA to pass through the gut lining into the bloodstream. Pattern recognition receptors such as TLRs and NLRs recognize these microbial components and activate inflammatory signaling pathways, including NF-κB and the NLRP3 inflammasome, leading to local and systemic inflammation. This dysfunctional, inflamed barrier can create a feedback loop between intestinal microbial imbalance and CKD. Abbreviations: CKD, chronic kidney disease; LPS, lipopolysaccharide; NF-κB, nuclear factor kappa B; NLR, nucleotide binding oligomerization domain like receptor; NLRP3, NLR family pyrin domain containing 3; SCFAs, short chain fatty acids; TLR, Toll-like receptor.

**Figure 4:**
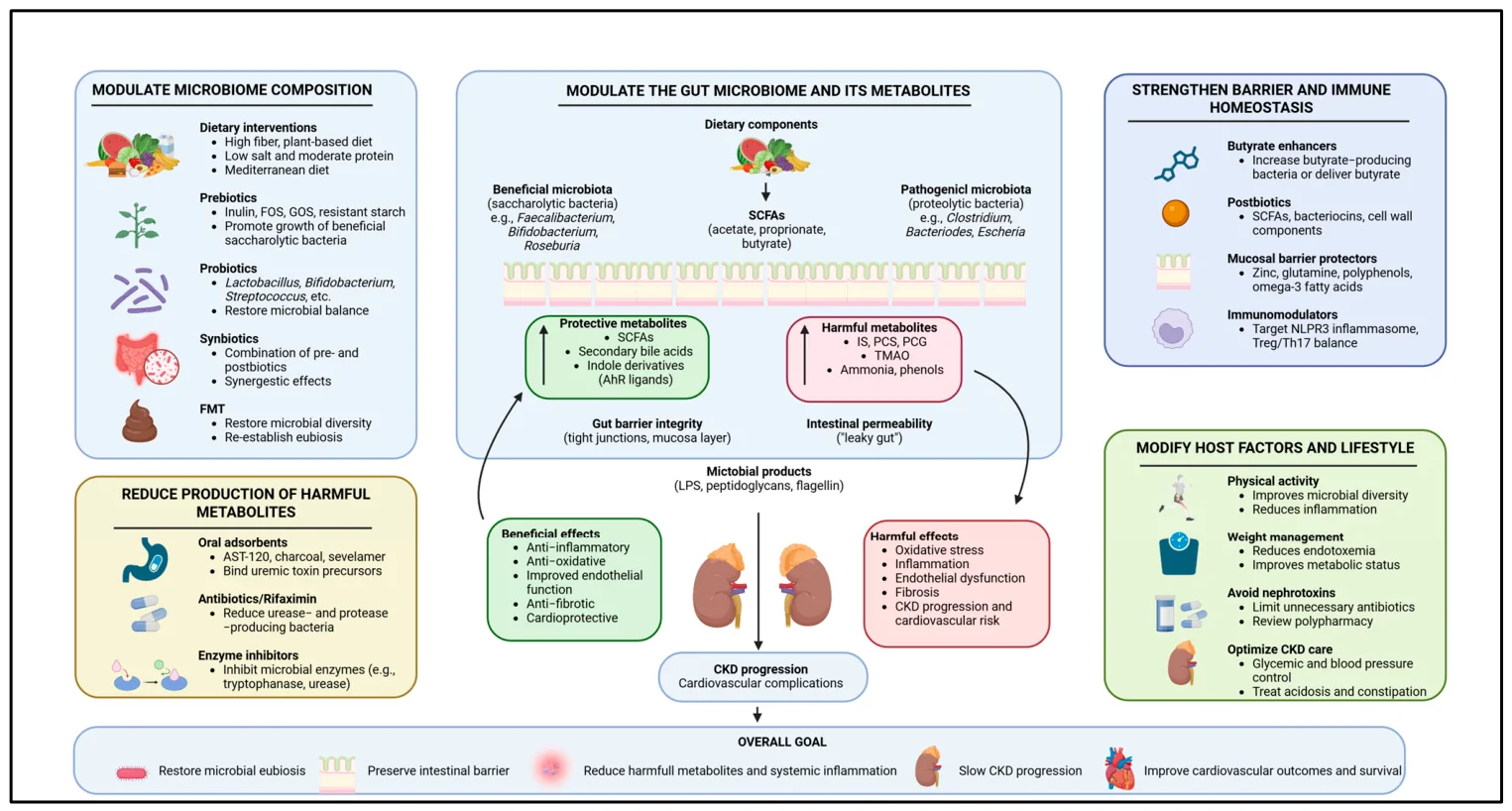
Therapeutic targets along the gut–kidney axis in CKD. This brief overview summarizes current and potential microbiome-targeted interventions at different levels of the gut–kidney axis. Current and emerging strategies include dietary modification, prebiotics, probiotics, synbiotics, fecal microbiota transplantation, oral adsorbents, selective inhibition of bacterial enzymes, postbiotics, and approaches to strengthen the intestinal barrier. Researchers are also exploring immunomodulation, lifestyle changes, and improved CKD management. Abbreviations: AhR, aryl hydrocarbon receptor; CKD, chronic kidney disease; FMT, fecal microbiota transplantation; FOS, fructooligosaccharides; GOS, galactooligosaccharides; IS, indoxyl sulfate; LPS, lipopolysaccharide; NLRP3, nucleotide-binding leucine-rich repeat receptor family pyrin domain-containing 3; PCG, p-cresyl glucuronide; PCS, p-cresyl sulfate; SCFAs, short-chain fatty acids; Th17, T helper 17; TMAO, trimethylamine N-oxide; Treg, regulatory T cell.

**Table 1:** Major alterations of the gut microbiota associated with chronic kidney disease and their functional consequences.

Taxon	Taxonomic Level	Reported Direction in CKD	Population/Model	Reported Association	Evidence Type	Consistency	Interpretation	Ref
*Enterobacteriaceae*	Family	Increased	Human CKD/terminal kidney failure cohorts	Higher abundance associated with CKD and inflammatory phenotype	Observational human	Variable across cohorts	Association, not evidence of causal CKD progression	[15,32]
*Faecalibacterium*	Genus	Decreased	Human CKD cohorts	Lower abundance and reduced representation of SCFA-producing taxa	Observational human	Reported in several cohorts	Potential functional relevance; causality unestablished	[15,32,33,34]
*Roseburia*	Genus	Decreased	Human CKD cohorts	Lower abundance associated with altered SCFA-related microbial function	Observational human	Variable	Association only	[15,32,33,34]
*Lactobacillus*	Genus	Variable	Human and experimental studies	Differences reported according to CKD stage and intervention	Mixed	Inconsistent	Direction cannot be generalized	[15,32,34]

Abbreviations: CKD, chronic kidney disease; SCFA, short-chain fatty acids.

**Table 2:** Major microbiota-related metabolites in CKD: origin, host handling, and level of evidence.

Metabolite	Microbial Precursor/Pathway	Host Metabolism	Renal Handling	Change Reported in CKD	Experimental Evidence	Human Evidence	Clinical Interpretation	Ref
IS	Tryptophan → indole	Hepatic oxidation and sulfation	Protein-bound; predominantly tubular secretion	Circulating concentrations increase with declining kidney function	Pro-inflammatory, oxidative and profibrotic effects demonstrated experimentally	Higher concentrations associated with adverse outcomes in observational studies	Accumulation reflects microbial generation and impaired renal elimination; causality in humans remains uncertain	[8,11,12,40,41,42,43,44]
PCS	Tyrosine/phenylalanine → p-cresol	Predominantly hepatic sulfation	Protein bound; tubular secretion important	Increased in advanced CKD	Experimental toxicity demonstrated	Observational associations with adverse outcomes	Human outcome evidence predominantly associative	[11,12,41,45,46,47]
PCG	p cresol	Hepatic glucuronidation	Renal elimination	Increased in CKD	Experimental biological effects reported	Human evidence more limited than for PCS	Clinical significance incompletely established	[48,49]
SCFAs	Fermentation of nondigestible carbohydrates	Local utilization and systemic metabolism	Not primarily considered retention solutes	Altered concentrations reported in CKD	Renoprotective effects in several experimental models	Predominantly observational human evidence	Therapeutic benefit of direct supplementation not established	[34,50,51,52,53,54,55,56]
TMAO	Dietary choline/carnitine → TMA	Hepatic FMO3 conversion	Strongly dependent on renal clearance	Increased in CKD	Experimental cardiovascular and renal effects reported	Associated with adverse outcomes	Elevated concentrations cannot be attributed solely to increased microbial generation	[57,58,59]

Abbreviations: CKD, chronic kidney disease; FMO3, flavin containing monooxygenase 3; IS, indoxyl sulfate; PCG, p-cresyl glucuronide; PCS, p-cresyl sulfate; SCFAs, short chain fatty acids; TMA, trimethylamine; TMAO, trimethylamine N-oxide.

**Table 3:** Summary of evidence for microbiome-targeted therapeutic strategies in chronic kidney disease. Evidence categories are descriptive and were not derived from a formal evidence-grading system. Preclinical evidence refers to in vitro and animal studies; human surrogate or biomarker evidence includes changes in microbiota composition, microbial metabolites, inflammatory markers, and other intermediate endpoints; and clinical outcome evidence refers to kidney function, kidney failure, cardiovascular events, hospitalization, or mortality. Evidence derived from nondialysis CKD and dialysis populations should not be considered interchangeable because differences in kidney function, uremic milieu, dietary exposure, medication use, and dialysis-related factors may modify both the microbiome and treatment response.

Intervention	Proposed Mechanism	Preclinical Evidence	Human Surrogate or Biomarker Evidence	Kidney Function and Kidney Failure Outcomes	Cardiovascular and Mortality Outcomes	Current Interpretation	Ref
Dietary interventions	Increased saccharolytic fermentation and SCFA production; reduced generation of PBUT precursors	Experimental support for effects on microbial metabolism and intestinal barrier function	Changes in microbiota composition, PBUTs and inflammatory markers reported	Effects on eGFR are inconsistent; kidney failure outcomes inadequately studied	No adequately powered evidence demonstrating reduced cardiovascular events or mortality	Biomarker effects do not establish disease modification	[82,83,84,88,89,91]
Prebiotics	Selective stimulation of saccharolytic bacteria and SCFA production; reduced proteolytic fermentation	Reduced PBUTs, inflammation and renal injury in experimental CKD	RCTs report variable reductions in IS, PCS, BUN and inflammatory markers	Little or no consistent improvement in eGFR or CKD progression	Cardiovascular and mortality outcomes have not been adequately evaluated	Evidence predominantly limited to surrogate outcomes	[101,102,104,105,106]
Probiotics	Modification of microbial composition and metabolism; potential reduction in uremic toxin precursor generation	Reduced PBUTs, inflammation, oxidative stress and renal fibrosis in experimental models	Small and heterogeneous effects on IS, PCS, BUN and inflammatory markers	No adequately powered evidence demonstrating sustained improvement in kidney function or reduced kidney failure	Cardiovascular and mortality outcomes have not been adequately evaluated	Preclinical effects have not translated into demonstrated clinical benefit	[105,113,114,115]
Synbiotics	Combined microbial supplementation and provision of fermentable substrates	Improvements in microbial metabolism, SCFA production and renal injury reported experimentally	Some RCTs demonstrate changes in microbiota composition and reductions in PCS and other surrogate markers	No adequately powered evidence demonstrating benefit on CKD progression or kidney failure	Cardiovascular and mortality outcomes have not been adequately evaluated	Surrogate benefits require confirmation against clinical endpoints	[118,127,128]
Postbiotics	Delivery of inanimate microorganisms and/or their components that modulate host signaling	Biological effects demonstrated mainly in experimental studies	Human CKD evidence remains very limited	No adequately powered evidence evaluating kidney function or kidney failure outcomes	Cardiovascular and mortality outcomes have not been adequately evaluated	Experimental strategy requiring clinical validation	[129]
Microbiota-derived metabolites/SCFAs	Direct supplementation or modulation of beneficial microbial metabolites to influence intestinal barrier function, immune signaling, inflammation, and host metabolism	Direct SCFA administration has attenuated renal inflammation and injury in experimental kidney disease	Human CKD evidence is predominantly observational; altered SCFA concentrations and SCFA-producing taxa have been reported	No established evidence that direct SCFA administration improves kidney function or prevents kidney failure in humans	No established evidence of cardiovascular or mortality benefit	Promising mechanistic strategy, but direct therapeutic evidence remains predominantly preclinical	[32,133,134,141]
AST-120	Intestinal adsorption of indole and other precursors, reducing systemic exposure to IS	Reduced IS, oxidative stress, inflammation and renal fibrosis in experimental CKD	Reduction in IS and selected surrogate markers demonstrated	Large randomized EPPIC trials did not demonstrate significant benefit on the primary composite kidney endpoint	No adequately powered evidence demonstrating cardiovascular or mortality benefit	Important example of biomarker modification failing to translate consistently into clinical benefit	[145,151,153,154]
FMT	Broad restructuring of the intestinal microbial ecosystem and its metabolic functions	Microbiota transfer experiments support causal effects of dysbiosis in experimental CKD	Very limited human evidence, mainly microbiome, metabolic, inflammatory and feasibility outcomes	No adequately powered evidence evaluating effects on eGFR decline or kidney failure	Cardiovascular and mortality outcomes have not been adequately evaluated	Experimental in CKD and should remain restricted to research settings	[162,163,164,165,166,167]
Exercise and lifestyle interventions	Potential modification of microbial diversity, SCFA production, inflammation and host metabolism	Mechanistic and experimental rationale	CKD-specific human microbiome data are limited	Microbiome-mediated effects on CKD progression have not been established	Microbiome-mediated cardiovascular or mortality benefit has not been established	Insufficient evidence to attribute established benefits of exercise specifically to microbiome modulation	[177,186]
Precision microbiome approaches	Individualized targeting based on metagenomic, metabolomic and host characteristics	Conceptual and emerging experimental evidence	No established biomarker efficacy in CKD	No clinical outcome evidence	No clinical outcome evidence	Research strategy rather than an established therapeutic intervention	[8,16,117,121,187]

Abbreviations: BUN, blood urea nitrogen; CKD, chronic kidney disease; eGFR, estimated glomerular filtration rate; FMT, fecal microbiota transplantation; IS, indoxyl sulfate; PBUTs, protein-bound uremic toxins; PCS, p-cresyl sulfate; RCT, randomized controlled trial; SCFA, short-chain fatty acid.

## Data Availability

No new data were created or analyzed in this study. Data sharing is not applicable to this article.

## Abbreviations

The following abbreviations are used in this manuscript:

AhR	aryl hydrocarbon receptor
AKI	acute kidney injury
AMPK	adenosine monophosphate-activated protein kinase
BCRP	breast cancer resistance protein
BUN	blood urea nitrogen
CKD	chronic kidney disease
CRP	C-reactive protein
CTGF	connective tissue growth factor
DKD	diabetic kidney disease
ECM	extracellular matrix
eGFR	estimated glomerular filtration rate
EMT	epithelial-to-mesenchymal transition
FMO3	flavin-containing monooxygenase 3
FMT	fecal microbiota transplantation
FOS	fructooligosaccharides
FXR	farnesoid X receptor
GLP-1 RA	glucagon-like peptide-1 receptor agonist
GOS	galactooligosaccharides
GPR	G protein-coupled receptor
HA	hippuric acid
HDAC	histone deacetylase
IAA	indole-3-acetic acid
ICAM-1	intercellular adhesion molecule-1
IL	interleukin
IS	indoxyl sulfate
ISAPP	International Scientific Association for Probiotics and Prebiotics
LPD	low-protein diet
LPS	lipopolysaccharide
MCP-1	monocyte chemoattractant protein-1
MRA	mineralocorticoid receptor antagonist
MRP4	multidrug resistance-associated protein 4
NADPH	nicotinamide adenine dinucleotide phosphate
NF-κB	nuclear factor kappa B
NLR	nucleotide-binding oligomerization domain-like receptor
NLRP3	NLR family pyrin domain containing 3
NO	nitric oxide
NOX	NADPH oxidase
OAT	organic anion transporter
PAGln	phenylacetylglutamine
PAI-1	plasminogen activator inhibitor-1
PAMP	pathogen-associated molecular pattern
PBUT	protein-bound uremic toxin
PCG	p-cresyl glucuronide
PCS	p-cresyl sulfate
PEW	protein-energy wasting
PTH	parathyroid hormone
RAAS	renin–angiotensin–aldosterone system
RCT	randomized controlled trial
ROS	reactive oxygen species
SCFA	short-chain fatty acid
SGLT2	sodium–glucose cotransporter 2
TGF-β	transforming growth factor-beta
TGR5	Takeda G protein-coupled receptor 5
TLR	Toll-like receptor
TMA	trimethylamine
TMAO	trimethylamine N-oxide
TNF-α	tumor necrosis factor-alpha
Treg	regulatory T cell
VCAM-1	vascular cell adhesion molecule-1
VLPD	very-low-protein diet
